# Disruption of *ArhGAP15* results in hyperactive Rac1, affects the architecture and function of hippocampal inhibitory neurons and causes cognitive deficits

**DOI:** 10.1038/srep34877

**Published:** 2016-10-07

**Authors:** Valentina Zamboni, Maria Armentano, Gabriella Sarò, Elisa Ciraolo, Alessandra Ghigo, Giulia Germena, Alessandro Umbach, Pamela Valnegri, Maria Passafaro, Valentina Carabelli, Daniela Gavello, Veronica Bianchi, Patrizia D’Adamo, Ivan de Curtis, Nadia El-Assawi, Alessandro Mauro, Lorenzo Priano, Nicola Ferri, Emilio Hirsch, Giorgio R. Merlo

**Affiliations:** 1 Department of Molecular Biotechnologies and Health Sciences, University of Torino, Italy; 2 Institute for Neuroscience, CNR Milano, Italy; 3 Department of Drug Science, University of Torino, Italy; 4 IRCSS San Raffaele Scientific Institute and San Raffaele University, Division of Neuroscience, Milano, Italy; 5 Department of Neurosciences, University of Turin & Div. of Neurology and Neurorehabilitation, S.Giuseppe Hospital, Istituto Auxologico Italiano IRCCS, Piancavallo (VB), Italy; 6 Department of Pharmaceutical and Pharmacological Science, University of Padova, Italy

## Abstract

During brain development, the small GTPases Rac1/Rac3 play key roles in neuronal migration, neuritogenesis, synaptic formation and plasticity, via control of actin cytoskeleton dynamic. Their activity is positively and negatively regulated by GEFs and GAPs molecules, respectively. However their *in vivo* roles are poorly known. The *ArhGAP15* gene, coding for a Rac-specific GAP protein, is expressed in both excitatory and inhibitory neurons of the adult hippocampus, and its loss results in the hyperactivation of Rac1/Rac3. In the CA3 and dentate gyrus (DG) regions of the *ArhGAP15* mutant hippocampus the CR+, PV+ and SST+ inhibitory neurons are reduced in number, due to reduced efficiency and directionality of their migration, while pyramidal neurons are unaffected. Loss of *ArhGAP15* alters neuritogenesis and the balance between excitatory and inhibitory synapses, with a net functional result consisting in increased spike frequency and bursts, accompanied by poor synchronization. Thus, the loss of *ArhGAP15* mainly impacts on interneuron-dependent inhibition. Adult *ArhGAP15*^−/−^ mice showed defective hippocampus-dependent functions such as working and associative memories. These findings indicate that a normal architecture and function of hippocampal inhibitory neurons is essential for higher hippocampal functions, and is exquisitely sensitive to *ArhGAP15*-dependent modulation of Rac1/Rac3.

The small GTPases of the Rho family, comprising Rho1, Rac1/3 and cdc42, tightly control the dynamic and re-organization of the actin cytoskeleton, an activity at the basis of neuronal migration, neuritogenesis and spine formation^1^,^2^. Rac GTPases govern lamellipodium and membrane ruffle formation, coordinate actin polymerization and microtubule stability and contribute to determine cell polarity in many cell types, including cortical and hippocampal neurons^1^,^3^,^4^.

Rac1 is highly and widely expressed in the embryonic and adult brain, while Rac3 is expressed at low levels in the developing nervous system^3^. Rac1/3 have been shown to participate in nearly all steps of neuronal genesis, maturation and circuit formation, including migration, neuritogenesis and spine formation, *in vitro* and *in vivo*^3^,^4^,^5^,^6^,^7^. While the full disruption of *Rac1* causes early embryonic lethality, conditional deletion of *Rac1* the brain results in axon guidance and radial migration defects^8^, while *synapsin1-cre*-mediated conditional deletion of *Rac1 (Rac1N*) in post-mitotic neurons leads to subtle migration, differentiation and connectivity defects affecting hippocampal inhibitory neurons (interneurons, INs) and hilar mossy cells^9^,^10^,^11^. The single disruption of *Rac3* causes mild histoanatomical and cognitive deficits^12^, however the combined *Rac1N* and *Rac3* null mutations result in aggravated defects of migration and circuit organization of cortical and hippocampal INs^3^,^11^, as well as altered migration and connectivity of hilar mossy cells^9^,^10^,^13^. The impact of Rac-GTPases on INs is increasingly being recognized. Migration, morphology and activity of cortical and hippocampal INs are strongly affected by the combined *Rac1N*^+*/−*^ and *Rac3*^−/−^ mutations: hippocampal and DG principal cells are hyperexcitable and mice show spontaneous epilepsy, due to IN dysfunctions^11^. Globally, these phenotypes suggest a model in which Rac1/3 activity is required for a positive regulation of cytoskeletal dynamic during migration, neuritogenesis and synaptogenesis. In turn, Rac1/3 activity responds to time- and region-restricted signals, most likely downstream of phosphinositide 3-kinase (PI3K)-mediated membrane signalling^14^,^15^.

Rho GTPases function as a binary switch cycling between an inactive GDP-bound form and an active GTP-bound state. This process is tightly regulated by guanine nucleotide exchange factors (GEFs), GTPase-activating proteins (GAPs) and guanine nucleotide dissociation inhibitors (GDIs)^16^. ArhGAP15 is a negative regulator of Rac1 activity, and its over-expression results in increased actin stress fibers and cell contraction. ArhGAP15 is structurally and phylogenetically related to ArhGAP9 and ArhGAP12, with which it shares a highly conserved Rac1-binding motif^17^,^18^. ArhGAP15 comprises a RhoGAP domain and a pleckstrin homology domain which mediates the peripheral localization and consequent activation of ArhGAP15, via binding to the PI3K product phosphatidylinositol 3,4,5-trisphosphate^18^. The GAP domain binds the C-terminal half of Rac1 in a nucleotide-independent manner and promotes the GDP-bound state, with a consequent inactivation of the downstream pathway. Both the PH and the GAP activities are essential for ArhGAP15 to exert its effect on cytoskeleton organization.

Given the control exerted by GAPs and GEFs over the activity of small GTPases, not surprisingly these modulators control many aspects of neuronal maturation, from morphology and polarity, to migration, neuritogenesis, spine and synaptic plasticity and even axon pruning^19^,^20^,^21^,^22^,^23^. Importantly, genes coding for proteins of the Rho/Rac GTPases pathways and their modulators, but not the GTPases themselves, are mutated in hereditary forms of intellectual disability (ID) in human, and affect neuritogenesis and spine/synapse morphology and dynamics^19^,^24^,^25^,^26^,^27^,^28^. For instance *Oligophrenin-1 (OPHN1*) is mutated in non-syndromic X-linked ID, and codes for a GAP that modulates RhoA activity and controls spine morphogenesis^19^,^25^,^29^. The *PAK3* gene, coding for an effector protein downstream of the small-GTPases, is mutated in X-linked ID, and the mutation impairs actin dynamics in dendritic spines^30^. The gene *PAK-interacting exchange factor (α-PIX*), also known as *ARHGEF6*, is mutated in certain forms of X-linked ID^31^. The *α*-Pix protein is an upstream activator of RhoA, and possibly Rac1/Rac3, and its mutation results in hypoactive Rac1 and defects in synaptic plasticity^24^,^28^. Thus, in human, both hyper- and hypo-activation of Rho-GTPases, due to mutations of regulatory proteins, are directly linked to ID via altered synaptic networks and plasticity^21^. Loss of *ArhGAP15* has been documented in a rare variant of the Mowat-Wilson disease, characterized by severe neurological deficits, severe ID, speech impairment and autism^32^,^33^. The loss of *ArhGAP15* accompanies the loss of the recognized disease gene *Zeb2*^34^, nonetheless it might contribute to the severity of these conditions or, alternatively, *ArhGAP15* could act as a modifier gene.

Gaining a deep knowledge of the specific action of each GAP and GEF of the Rho/Rac GTPases for neuronal migration, neuritogenesis and synaptogenesis is of considerable importance; however much of our current knowledge is derived from the use of either dominant-negative or constitutively active Rac1 mutant proteins, or from the analysis of mice with conditional *Rac1/3* loss-of-function mutations^6^,^7^,^9^,^10^,^11^. However, genetic models of *Rac1/3* loss-of-function may not fully elucidate the role of these GTPases in terms of misregulated activity; for instance, little is known about the effect of hyperactivation of Rac1/3, *in vivo*. Here we address this question by examining the consequence of hyperactive Rac1/3 resulting from the depletion of *ArhGAP15* in mice, in terms of neuronal migration, differentiation, organization, electrical functions and behavioural performance. We found that loss of *ArhGAP15* alters neuritogenesis and the synaptic balance between excitatory and inhibitory synapses. Overall specific populations of interneurons (INs) are most affected, with altered migration and number, resulting in reduced inhibition and synchronicity. *ArhGAP15*^−/−^ mice show behavioural deficits similar to those seen in mice with *Rac1/3* loss-of-function mutations.

## Results

### Loss of *ArhGAP15* results in hyperactivation of Rac1/3

**Figure 1:**
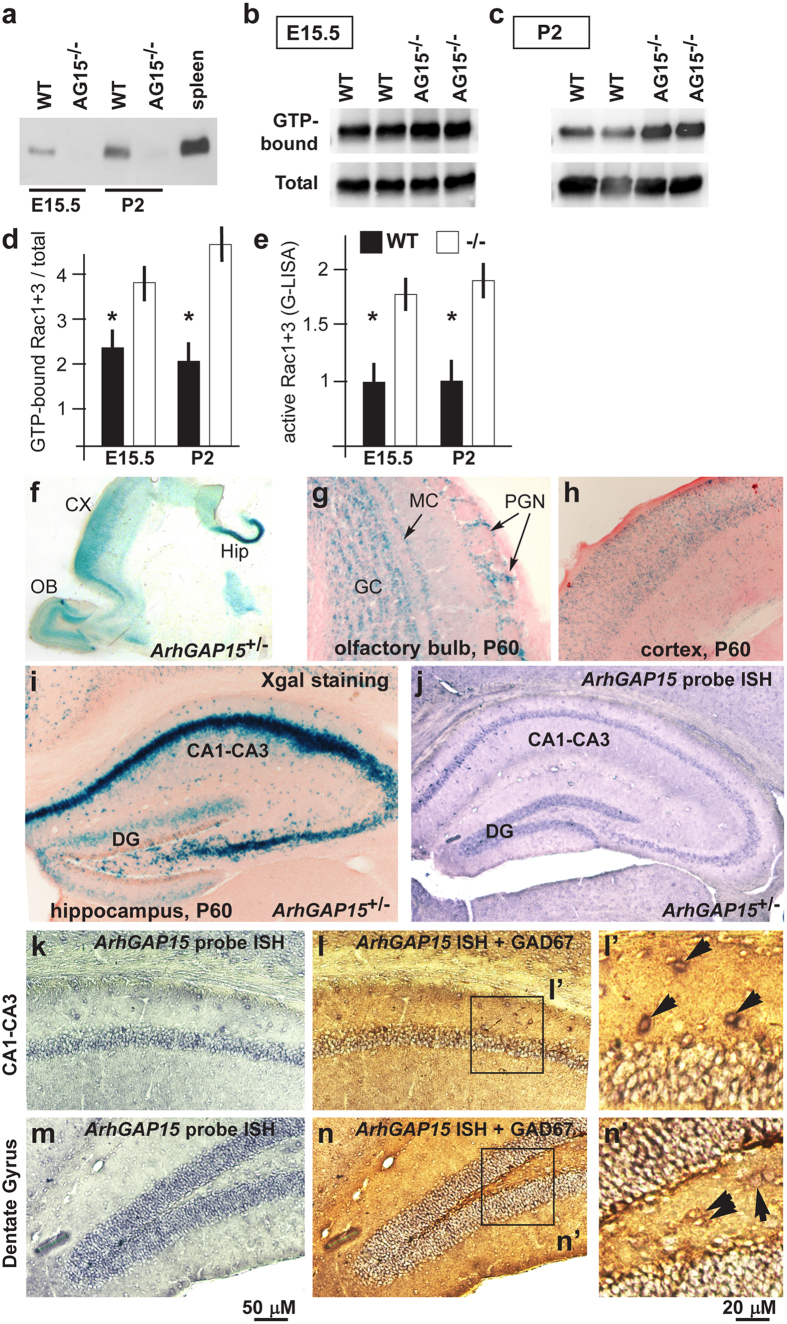
Expression of *ArhGAP15* in the mouse hippocampus. (**a)** Western blot analysis of samples immunoprecipitated from total protein extracts of WT and *ArhGAP15* null brains, at the indicated ages. As control, WT and *ArhGAP15* null macrophages were used. (**b,c)** Rac1 activity in whole-forebrain protein lysates from E15.5 (**b**) or from P2 (**c**) animals, with WT or *ArhGAP15*^−/−^ genotype (indicated on the top), by pull-down assay, followed by Western blot analyses. GTP-bound Rac (top lanes) and total Rac (bottom lanes) are shown. Samples represent biological replicates. (**d**) Quantification of the pull-down/Western assays on the left, using total Rac for normalization. (**e)** Rac1+ Rac3 activity measured by G-LISA, on embryonic (E15.5) and neonatal (P2) whole-forebrain protein lysates from WT and *ArhGAP15*^−/−^ animals. *Indicates p < 0.05. **(f**) Xgal staining of longitudinal sections of *ArhGAP15*^+*/−*^ brain, at the age P2. The olfactory bulb (OB), cortex (CX) and hippocampal (HIP) are indicated. (**g,h**) Xgal staining of coronal sections of *ArhGAP15*^+*/−*^ brain, at the age P60, corresponding to the OB (**g**) and the somatosensory cortex (**h**) at low magnification. The granule cells (GC), the mitral cells (MC) and the periglomerular neurons (PGN) are indicated. (**i)** Xgal staining of coronal sections of adult *ArhGAP15*^+*/−*^ brain, counterstained with eosin. (**j)** *In situ* hybridization with a probe detecting *ArhGAP15* mRNA on coronal sections of adult WT hippocampi. **(k–n**) *In situ* hybridization detecting *ArhGAP15* mRNA on coronal sections of WT adult CA1–CA3 (**k**) and DG (**m**) regions, followed by immunostaining with anti-GAD67 (**l,n**). On the right (l’ and n’), higher magnification from l and n of double positive (*ArhGAP15* mRNA+ GAD67) neurons (black arrows). Scale bars are reported in panels l and n’.

We immunoprecipitated protein extracts from WT and *ArhGAP15*^−/−^ mouse brains, with anti-ArhGAP15 antibody. A protein of the expected molecular weight (54 KD) was detected in samples from E15.5 and early postnatal (P2) WT animals, while no signal of the corresponding size was observed in samples from *ArhGAP15*^−/−^ animals (Fig. 1a). As positive control, expression was verified in the spleen^18^.

To determined the effect of the absence of ArhGAP15 on Rac1 activity, we used a pull-down assay, as described^18^. In samples from postnatal (P2) *ArhGAP15*^−/−^ brains the fraction of GTP-bound Rac1/3 was 2-fold higher than in WT samples, while in samples from E15.5 embryonic brains GTP-bound Rac1/3 was about 1.7-fold higher than in mutant samples (Fig. 1b–d). However the MAb clone 23A8 (Upstate Biotech) used for pull-down (data not shown), as well as the antibodies currently available (de Curtis, personal communication), do not discriminate Rac1 from Rac3, due to the high similarity. We therefore used a second and independent test, consisting in a G-LISA assay for Rac1/3 followed by colorimetric determination of the active fraction, and we measured a 1.8 folds activation of Rac1/3 in the mutant samples (Fig. 1e). However, also the G-LISA assay does not discriminate Rac3 from Rac1.

### Expression of *ArhGAP15* in hippocampus

We examined the expression of the *lacZ* knock-in reporter in sections of *ArhGAP15*^+*/−*^ brains by Xgal staining. No expression was observed prior to E13.5. At E14.5 and E15.5 we detected expression in the cortical and hippocampal primordia (data not shown). No Xgal staining is observed in the GE and in regions of proliferating neuroblasts, at the age E14–E15. Xgal staining of early postnatal (P2) and adult (P60) *ArhGAP15*^+*/−*^ brains showed signal in the olfactory bulbs, neocortex and hippocampus (Fig. 1f–h). In the hippocampus, expression is observed in the pyramidal layer of the CA1–CA3 regions and in the dentate gyrus (DG) (Fig. 1i). Considering that the βgal protein is highly stable and may persist in βgal-negative cells, we examined the expression of the *lacZ* mRNA by *in situ* hybridization. The localization of the *lacZ* mRNA overlapped that of Xgal staining (Supplementary Fig. S1).

Next we carried out RNA:RNA *in situ* hybridization with a probe detecting *ArhGAP15* mRNA on coronal sections of WT brains, and obtained a distribution identical to the Xgal staining and the *lacZ in situ* hybridization, done on *ArhGAP15*^+*/−*^ brains (Fig. 1j). Expression of *ArhGAP15* was evident in the pyramidal cell layer of the CA1–CA3 regions and in the granule neurons of the DG. To verify expression in INs, we combined *in situ* hybridization for *ArhGAP15* mRNA (blue) with immunostaining for GAD67 (brown) on the same sections. A large fraction of GAD67+ cells were also positive for hybridization with the *ArhGAP15* probe (Fig. 1k–n,l’). We repeated this using Xgal staining of coronal sections of the hippocampus from *ArhGAP15*^+*/−*^ animals, followed by immunostaining with anti-GAD67 antibody (brown and blue, respectively). Double staining was detected in a large fraction (>85%) of hippocampal and DG neurons (Supplementary Fig. S1), indicating that *ArhGAP15* is expressed in most pyramidal cells and GABAergic INs.

### Organization of hippocampal INs in the absence of *ArhGAP15*

Xgal staining of *ArhGAP15*^+*/−*^ and ^−/−^ mice did not reveal differences in distribution of *ArhGAP15/lacZ* -expressing cells, indicating that these cells are not lost or mislocalized in the absence of *ArhGAP15*. To better verify this we estimated the number of pyramidal and granule neurons in the CA1–CA3 and the DG regions, respectively, comparing WT and *ArhGAP15*^−/−^ brains. We carried out immunostaining to detect NeuN, and counterstained the sections with DAPI to determine the total number of nuclei. The number of NeuN+ nuclei in the pyramidal cell layer of CA1–CA3 regions and in the granule cell layer of the DG was unchanged in the *ArhGAP15*^−/−^ specimen (Supplementary Fig. S2). Likewise, we determined the number and position of inhibitory neurons and astrocytes, by immunostaining for GAD67+ and GFAP+, respectively, in the same regions. While the number of astrocytes in *ArhGAP15*^−/−^ specimens was unchanged, the number of GAD67+ neurons was diminished in the DG and CA3 regions of mutant brains, while did not change in the CA1 and CA2 regions (Supplementary Fig. S3). Thus, in *ArhGAP15*^−/−^ mice the number, position and marker expression of excitatory neurons and astrocytes are not grossly altered, while the number of INs is reduced in specific areas.

**Figure 2:**
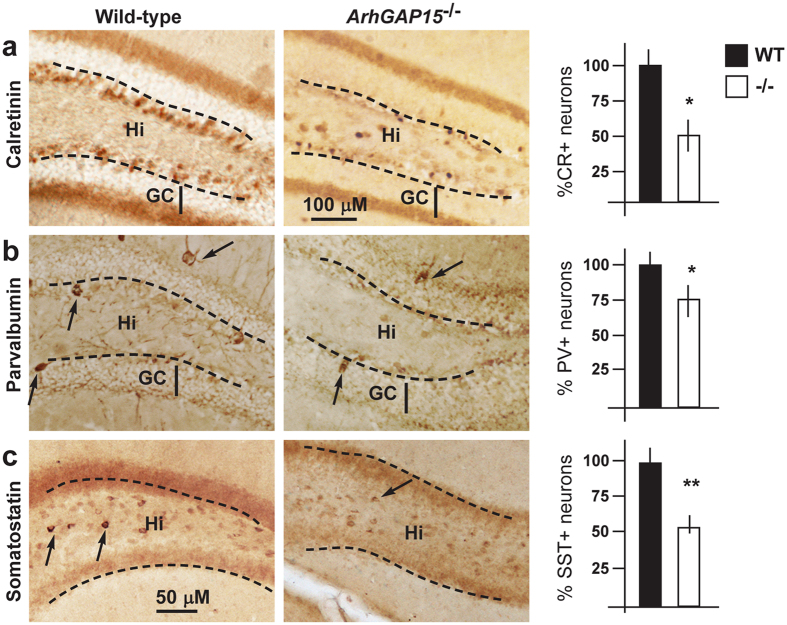
Hippocampal interneurons in the *ArhGAP15*^−/−^ brain. (**a–c**) Representative immunostaining of coronal sections of adult DG from WT (left) and *ArhGAP15*^−/−^ (right) animals, with anti-CR (**a**), anti-PV (**b**) and anti-SST (**c**). Positive neurons are indicated with black arrows. The borders between the hilus (Hi) and the granule cell (GC) are indicated with dashed lines. On the right, quantification of the number of positive neurons in the DG region. The WT samples (N = 6) were set = 100%, indicated with solid bars. *ArhGAP15*^−/−^ samples (N = 3) are indicated with open bars. Asterisks indicate significance (*Indicates p < 0.05; **Indicate p < 0.01). Scale bars are reported in panels a and c.

We examined in detail the number and distribution of IN subtypes within the adult hippocampus, by immunostaining coronal sections for CR+, PV+ and SST+, the three main classes of INs in these regions. In the DG of *ArhGAP15*^−/−^ brains we observed a reduced number of CR+ (−42%), PV+ (−20%) and SST+ (−45%) neurons (Fig. 2a–c). Staining of hippocampus sections from adult (P30) *ArhGAP15*^+*/−*^ and ^−/−^ brains for apoptotic cells, by the TUNEL method, did not reveal significant changes in the number and position of TUNEL+ cells (data not shown), thus we exclude a loss of specific IN populations due to apoptosis. No difference was observed in the number of CR+, PV+ and SST+ neurons in the CA1–CA3 regions, although the overall number of GAD67+ was slightly reduced; this might be due to lack of other IN subtypes.

### Tangential migration of hippocampal INs in the absence of *ArhGAP15*

**Figure 3:**
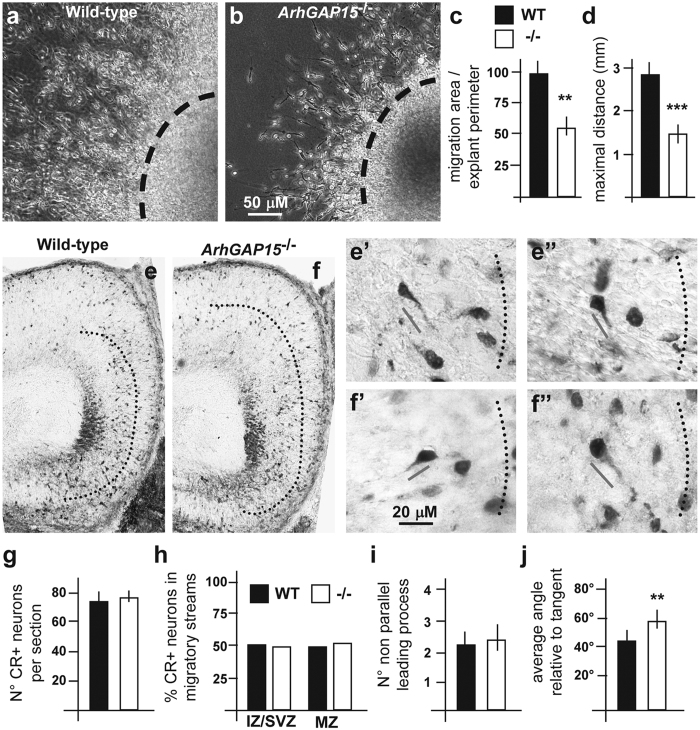
Tangential IN migration in the embryonic *ArhGAP15*^−/−^ hippocampus. (**a,b**) Young INs out-migrating from explants of hippocampal primordia of WT (**a**, N = 4) or *ArhGAP15*^−/−^ (**b**, N = 3) E17.5 embryos, maintained 3 DIV. (**c,d)** Quantification of the efficiency of outmigration, by determining the area occupied by migrating cell over the explant perimeter (**i**) and the maximal distance travelled (at least 80 cells) (**j**). **Indicate p < 0.01; ***Indicate p < 0.001. (**e,f)** Immunostaining of coronal sections of WT (**e**, N = 3) or *ArhGAP15*^−/−^ (**f**, N = 3) hippocampal primordia at the age E17.5, with anti-CR, to detect tangentially migrating immature INs. (e’,e”,f’,f”) Individual CR+ cells from WT (e’,e”) or *ArhGAP15*^−/−^ (f’,f”) brains, with a visible leading process. The tangential orientation is indicated (dotted lines). Scale bars are reported in panels b and f’. (**g)** Total number of CR+ cells per section (20 sections counted, 3 animals per genotype). No difference is detected. (**h)** Number of CR+ neurons scored according to their pial-to-ventricular position, in the Marginal Zone (MZ) or in the Intermediate/Subventricular Zone (IZ/SVZ), expressed in percentage over the total. No difference is detected. (**i)** Number of CR+ cells with a leading process with an angle higher than 20 degree relative to the pial-ventricular (tangential) orientation. (**j)** Angle of the leading process versus the tangential direction of neurons showing a >20° deviation from the tangent; average of a minimum of 40 CR+ neurons for each genotype. A significant increase of the deviation is observed in the absence of *ArhGAP15*, compared to the WT controls (**p < 0.01).

A reduced number of INs in the DG of adult *ArhGAP15*^−/−^ mice could also result from altered tangential migration of immature INs from the hippocampal primordium, during development^35^. We assayed the efficiency of emergence of young neurons from explants of hippocampal primordium *in vitro*, comparing E17.5 WT with *ArhGAP15*^−/−^ brains. Emerging neurons from *ArhGAP15* mutant explants occupied a smaller area (970 vs. 575 μm, p < 0.002) and reached a shorter maximal distance (1.56 vs. 2.9 mm, p < 0.0002), compared to the control explants (Fig. 3a–d). Immunostaining of cultured hippocampal explants for GAD67 revealed that a considerable fraction (15%) of out-migrated neurons, and in particular the longest migrating ones (70%), are of the inhibitory type (Supplementary Fig. S4). Thus the loss of *ArhGAP15* affects the migration efficiency of hippocampal INs.

Rac1 is required for the formation of the leading edge, which directs neuron migration^14^,^36^. Since Rac1/3 is hyperactive in the *ArhGAP15*^−/−^ brain, we examined the tangential migration of early hippocampus INs in *ArhGAP15*^−/−^ mice, by immunostaining coronal sections of E17.5 brains for CR, a marker of immature INs. We assessed the number and position of CR+ cells in the entorhinal cortex and in the hippocampal primordium, but no difference was observed either in the total number of CR+ neurons or in their distribution (Fig. 3e–h). Next we examined the orientation of the leading process of CR+ neurons relative to a direction parallel to the pial surface (the tangent), and compared WT and *ArhGAP15*^−/−^ E17.5 brains. First we determined the number of CR+ neurons showing an angle deviating >20° from the tangent, and observed no difference (Fig. 3i). Next we considered only those neurons showing a leading process oriented with an angle deviating >20° relative to the tangent, and examined at least 50 neurons per genotype; in the absence of *ArhGAP15* we detected a significant increase (41.4 ± 2.7° in WT; 54.5 ± 2.9° in *ArhGAP15*^−/−^; p = 0.003) of the mean angle with the tangent (Fig. 3j), indicating that immature *ArhGAP15*^−/−^ CR+ deviate more than the WT counterpart. Thus *ArhGAP15* participates in the control of directionality during tangential migration.

### Inhibitory and excitatory synapses in *ArhGAP15* ^−/−^ hippocampi

**Figure 4:**
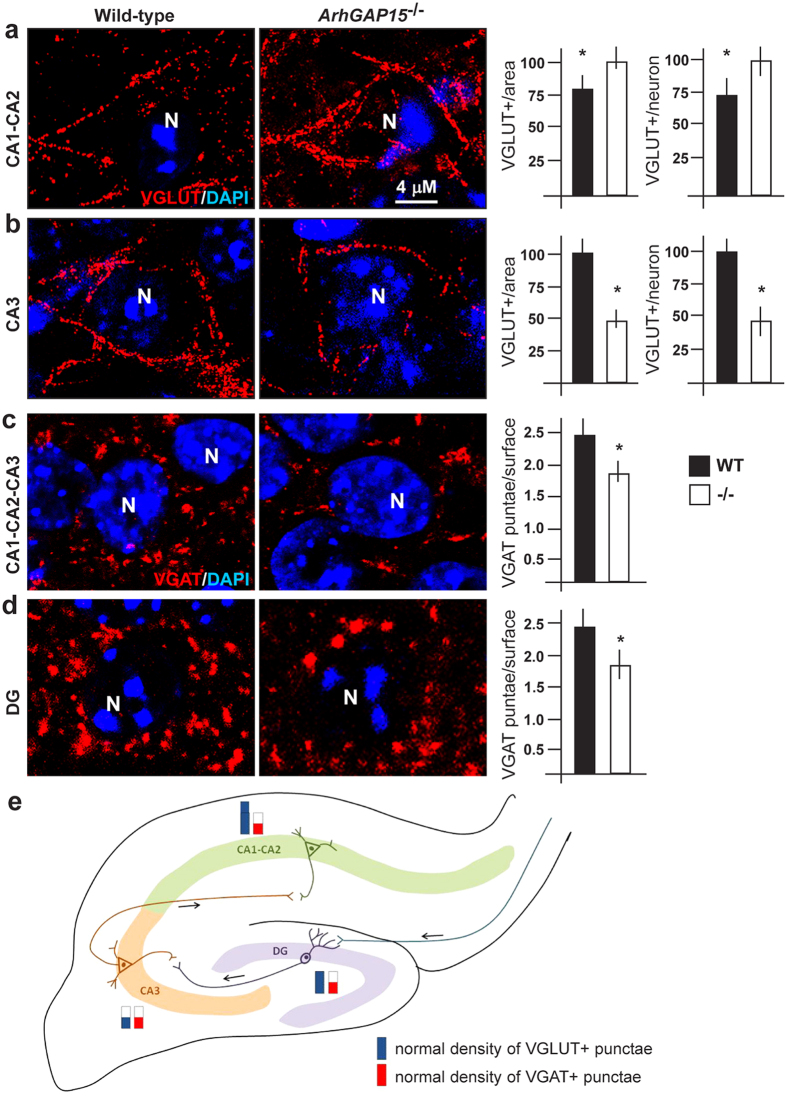
Density of excitatory and inhibitory synapses in *ArhGAP15*^−/−^ hippocampi. (**a,b**) Representative immunostaining of the CA1–CA2 (**a**) and the CA3 (**b**) regions of the hippocampus with anti-VGLUT (red fluorescence), counterstained with DAPI (blue). Left panels, WT, right panel, *ArhGAP15*^−/−^. The histograms on the right report the average number of VGLUT+ *punctae* per number of neuronal somas (>80 examined), and the density of the same per area examined, the CA1–CA2 and in the CA3 region. Three WT and five *ArhGAP15*^−/−^ brains were examined. In the DG no difference was detected. (**c,d)** Representative immunostaining of the CA1, CA2, CA3 (**c**) and the DG (**d**) regions with anti-VGAT (red fluorescence), counterstained with DAPI. The histograms on the right report the average number of VGAT+ *punctae* per surface of neuronal soma (>100 examines) in the CA1–CA3 or in the DG region. A significant reduction of VGAT+ *punctae* was also detected in all regions of the hippocampus. WT, solid bars, *ArhGAP15*^−/−^, open bars. Asterisks indicate statistical significance (p < 0.05). Scale bar is reported in panel a. (**e)** Diagram summarizing the changes in density of excitatory (VGLUT) and inhibitory (VGAT) synapses in various hippocampal regions of *ArhGAP15*^−/−^ brains. The CA1, CA2, CA3 and the DG regions are indicated with different colours. The red and blue bars represent, respectively, the density of excitatory (VGLUT) and inhibitory (VGAT) spines. The solid bars indicate the actual observation, the dashed faint bars indicate the normal value, relative to control brains.

We examined the status of excitatory synapses in various regions of the hippocampus by determining the density of VGLUT+ *punctae* on the perisomatic surface of pyramidal neurons in CA1–CA2 (projections from CA3 pyramidal neurons), in CA3 (projections from the granule neurons of the DG) and in DG (projections from the entorhinal cortex). We immunostained sections of *ArhGAP15*^−/−^ and control P30 brains with anti-VGLUT and determined the number of VGLUT+ *punctae* relative to: (a) number of neuronal bodies; (b) the area examined. The results indicate an increase of the density of excitatory synapses in the CA1–CA2 region (+24% relative to WT, p < 0.005), and a decrease in the CA3 region (−51% relative to WT, p < 0.002) (Fig. 4a,b). No difference was detected in the DG.

We then examined the inhibitory synapses. Given the altered number of INs in specific areas of the hippocampus, we determined the number of VGAT+ *punctae* on the perisomatic surface of pyramidal neurons in CA1–CA3, and of granule cells in the DG, by immunostaining of coronal sections of P30 brains for VGAT. In *ArhGAP15*^−/−^ specimens we observed a reduction of the density of VGAT+ *punctae* in the CA1, CA2 and CA3 regions (globally 2.5/10 μm vs. 3.5/10 μm, p < 0.002) and in the DG (2.6/10 μm vs. 3.6/10 μm, p < 0.005) regions (Fig. 4c,d). Thus, in CA1–CA2 the excitatory synapses prevail over the inhibitory synapses, while in CA3 both types are reduced in number (summarized in Fig. 4e). In the DG, the excitatory and inhibitory synapses are unbalanced in favour of excitation. These data point to an altered balance between excitation and inhibition.

### Neuritogenesis and morphology in the absence of *ArhGAP15*

**Figure 5:**
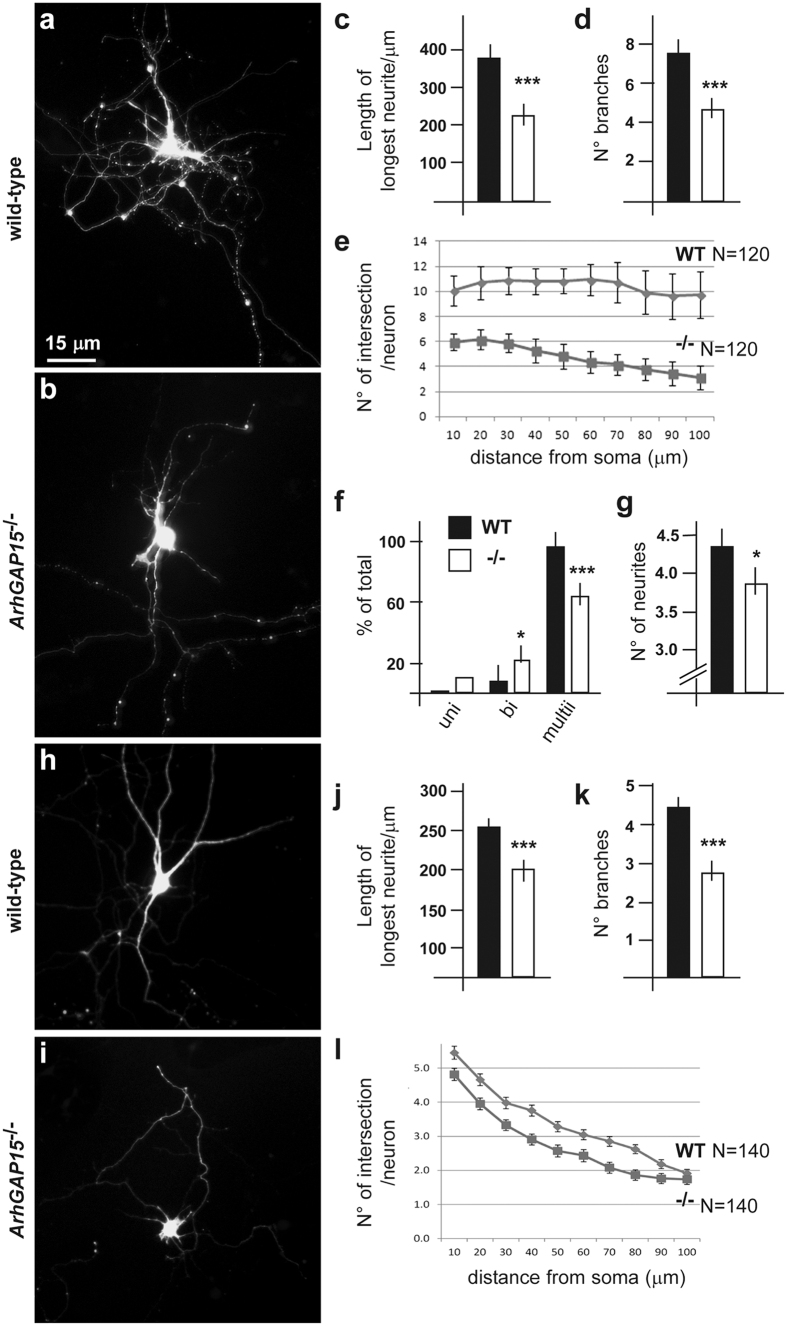
Neuritogenesis and polarity of cultured hippocampal and MGE neurons. (**a,b**) Representative micrographs of cultured hippocampal neurons transfected with a GFP-expression vector, after 7 DIV, to examine neuritogenesis in the presence (WT, **a**) or in the absence (**b**) of ArhGAP15. Most neurons were morphologically pyramidal. Scale bar is reported in panel a. (**c–e)** Quantification of the length of the longest neurite in μm (**e**), of the number of branches (secondary neurites) (**f**) and the mean number of intersections as a function of distance from the soma (Sholl analysis, in i), comparing neurons from WT (black bars) and *ArhGAP15*^−/−^ (open bars) brains. (**f,g)** Quantitative analysis of neuronal polarity, expressed and the number of unipolar, bipolar and multipolar neurons (**j**) and as the number of primary neurites in multipolar neurons (**k**), comparing neurons from WT (black bars) and *ArhGAP15*^−/−^ (open bars) brains. A total of 120 neurons were examined for each genotype. (**h,i)** Representative micrographs of cultured neurons from dissociated MGEs from wild-type (**h**) or *ArhGAP15*^−/−^ E14.5 embryos, after 7 DIV. A large fraction of these neurons were shown to be GAD67 immunoreactive. (**j–l)** Quantitative analyses on neurite length (**j**), branching (**k**) and overally complexity by Sholl analysis (**l**). A total of 140 neurons were examined for each genotype. *Indicates p < 0.05; ***Indicate p < 0.001.

Since Rac1 is essential for axon growth and guidance^37^, we determine whether the loss of *ArhGAP15* affects neuritogenesis, adopting primary cultures of dissociated embryonic brains, transfected with a *Green Fluorescent Protein (GFP)* expressing vector. In a first set of experiments, we cultured dissociated neurons from E17.5 hippocampi, known to comprise mostly pyramidal neurons. After 7 DIV, 120 GFP+ neurons were examined for several indicators of neuritogenesis (representative micrographs in Fig. 5a,b). *ArhGAP15*^−/−^ neurons show a significant decrease in the length of the longest neurite (p < 10^−6^) (Fig. 5c), in the number of secondary neurites (p < 10^−6^) (Fig. 5d) and in the complexity of the neuritic arborisation, measured as the mean N° of intersections as a function of the distance from the soma (Sholl analysis) (p < 2 × 10^−2^ at each point) (Fig. 5e). Finally we counted the number of neurons showing a unipolar, a bipolar or a multipolar morphology, and the number of primary neurites of the multipolar neurons. We observed a reduced number of multipolar neurons, and these showed a reduced number of primary neurites (Fig. 5f,g). Collectively, these data indicate an overall reduced efficiency of neurite elongation and branching, and a simpler morphology of pyramidal neurons in the absence of *ArhGAP15*.

In a second set of experiments, we derived primary cultures from the embryonic MGE at the age E14.5, known to comprise progenitors and early differentiated INs fated to migrate to the cortex and hippocampus. Immunostaining of these cultures for GAD67, after 7 DIV, indeed showed that a large fraction of cells are GAD67+, as expected (Supplementary Fig. S5). After 7 DIV, we examined 140 neurons from WT and *ArhGAP15*^−/−^ brains (representative photographs in Fig. 5h,i); mutant neurons showed a significantly reduced length of the longest neurite (p < 3 × 10^−3^), reduced number of branches (p < 10^−10^) and a reduced overall complexity (p < 5 × 10^−3^) (Fig. 5j–l). These data indicate that young inhibitory neurons from *ArhGAP15*^−/−^ mutant brains have a reduced ability to acquire neuritic complexity.

### Reduced spine density upon downmodulation of *ArhGAP15*

**Figure 6:**
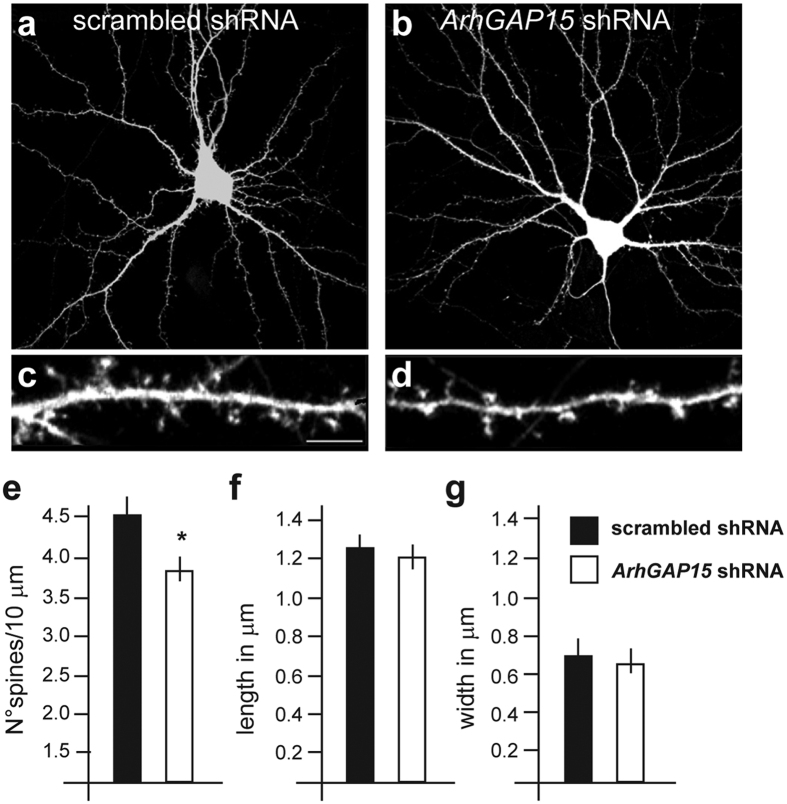
Spine density of hippocampal neurons upon downmodulation of *ArhGAP15.* (**a,b)** Representative images of dendritic spines of primary rat hippocampal neurons electroporated with a GFP-expressing vector, and treated with either a control (**c**) or an anti-*ArhGAP15* siRNA (**d**) oligonucleotide sequence. (**c,d)** Higher magnification of apical dendrites from **c** and **d**, respectively. Scale bar (=1 μm) is reported in panel c. (**e–g)** Histograms showing the quantification of spine density (**e**), length (**f**) and width (**g**) on the apical dendrites of pyramidal neurons transduced with control (solid bars) or with siRNA for *ArhGAP15* (open bars). A total of 40 neurons in three experiments was examined for each genotype. Density is expressed as number of spines per 10 μm of dendrite. A significant reduction of spine density is observed when endogenous *ArhGAP15* is silenced, while spine length and width were unchanged. *Indicates p < 0.05; ***Indicate p < 0.001.

We tested whether the absence of *ArhGAP15* may affect spinogenesis by adopting cultured rat E19 hippocampal neurons transduced with vectors expressing an anti-*ArhGAP15* shRNA sequence, previously shown to effectively deplete ArhGAP15 and result in increased levels of GTP-bound Rac1^18^. A scrambled shRNA sequence was used as control. This method was chosen to limit the analysis to the excitatory neurons, and to bypass developmental effects. Transduction with anti-*ArhGAP15* shRNA (GFP+ cells) resulted in reduced spine density (4.6 vs. 3.9 spines/10 μm), as compared to controls (Fig. 6). Conversely, spine length and width were unchanged upon silencing of *ArhGAP15*. The same cultures were stained for PSD95 and bassoon, to confirm proper synaptic organization, and for the GABAergic markers VGAT and GAD65 to confirm a normal distribution of inhibitory synapses. No differences in the staining with all these markers was detected (Supplementary Fig. S6), indicating that loss of *ArhGAP15* mainly affects the efficiency of spine formation. Finally, we carried out single-cell recordings of the same neurons transduced with anti-*ArhGAP15* to determine the membrane potential and the excitation threshold, but no difference was observed compared to controls (Supplementary Fig. S7).

### Electrical activity of *ArhGAP15* ^−/−^ cultured hippocampal neurons

**Figure 7:**
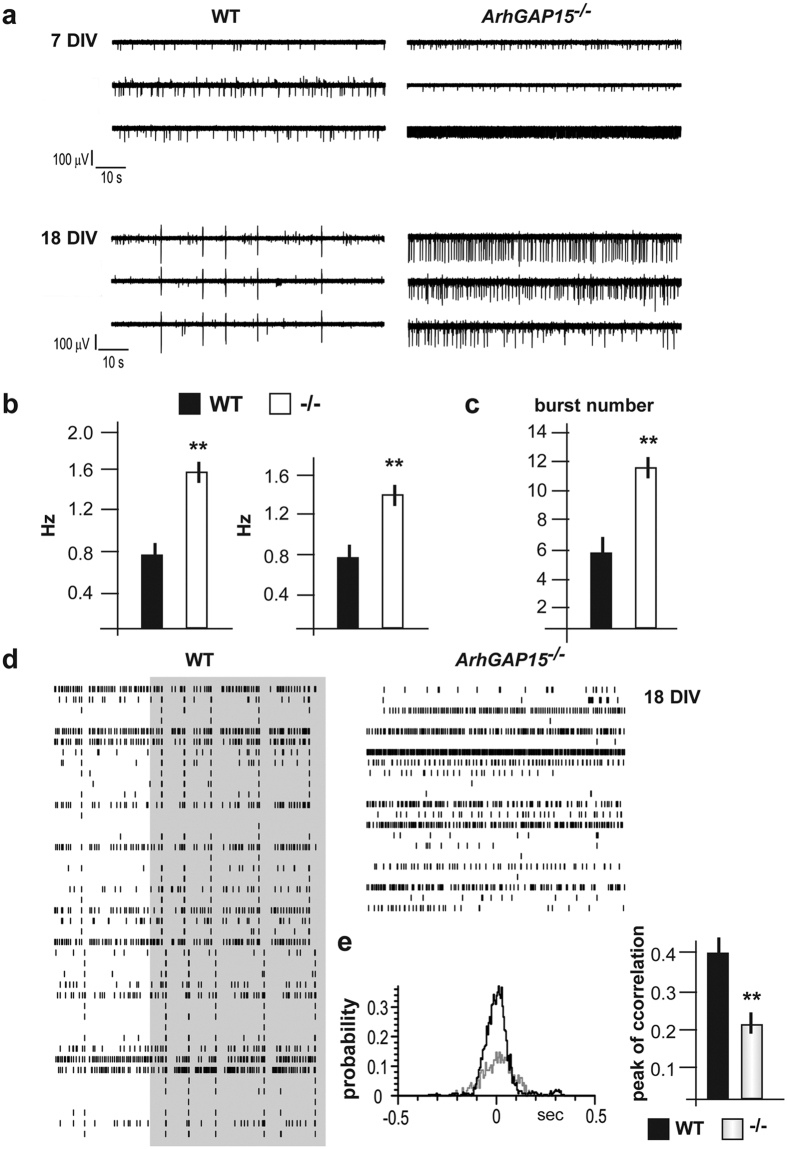
Electrical activity of primary neuronal cultures from *ArhGAP15*^−/−^ hippocampi. (**a)** Representative recordings (3 channels each) of cultures of late embryonic (E18) hippocampal neurons obtained from WT (left) or *ArhGAP15*^−/−^ embryos (right), maintained for 7 (top) or 18 (middle) days *in vitro* (DIV). (**b)** Quantification (three independent experiments) of the mean frequency at 7 (left) and 18 (right) DIV. (**c)** Quantification of the burst number at 18 DIV. In all cases, a significant increase is observed (asterisks). (**d)** Raster plots of the spontaneous activity of the hippocampal network from WT (left) or *ArhGAP15*^−/−^ embryos (right), at 18 DIV. The shaded area highlights synchronous activity. (**e)** Cross-correlogram plots showing the probability of coincidence of the events versus time (in seconds) of WT (black trace) and mutant (grey trace) cultures. The mean peak of correlation is reported in the histogram on the right. **Indicate p < 0.01.

The hippocampus and DG of adult *ArhGAP15*^−/−^ mice are equipped with a less complex neurite architecture, with altered excitatory and inhibitory balance and loss of specific IN subpopulations. To determine the functional consequence of this condition, we recorded the spontaneous electrical activity of primary cultures of hippocampal neurons, obtained from E17.5 WT or *ArhGAP15*^−/−^ brains, using multi-electrode arrays (MEA)^38^,^39^,^40^. First, we immunostained WT and mutant cultures with anti-GAD67 at 10 DIV and verified that these are composed of equal numbers of GABAergic neurons (data not shown). Next, we recorded the spontaneous activity at 7 and 18 DIV, and found that *ArhGAP15*^−/−^ cultures showed a similar onset of the activity, a 2-fold and a 1.5-fold increase in mean bursts frequency at 7 and 18 DIV, respectively, and a 2-fold increase in the overall burst number at 18 DIV (Fig. 7a). Young mutant cultures showed increased firing frequency with respect to WT (0.74 ± 0.11 Hz in WT; 1.5 ± 0.2 Hz in mutants; n = 10 MEAs, p < 0.001). With time in culture (18 DIV) both groups converted to bursting mode; mutant cultures exhibited increased firing frequency (0.85 ± 0.05 Hz in WT; 1.23 ± 0.16 Hz in mutants; n = 8 and n = 4 MEAs respectively, p < 0.01) and bursts number (6.0 ± 0.4 in WT; 11.6 ± 1.6 in mutants; n = 4 MEAs, p < 0.001), with a tendency to be more active and with signals distributed more randomly with respect to WT (Fig. 7a–c). To compare the network synchronization in WT versus *ArhGAP15*^−/−^, raster plots are shown in Fig. 7d: each action potential is represented as a vertical line and plotted versus time. At 18 DIV, the mutant cultures (right) are less synchronized then the WT controls (left) (Fig. 7d, grey rectangle). Synchronization was quantified as the probability of coincidence of single events between electrodes, computing cross-correlation histograms between a reference electrode and the remaining ones. During basal firing at different time windows (±0.5 s and ±3.5 s with 5 ms bin size) the maximal correlation of neuronal activities at t = 0 s is increased by 48% at DIV 18 (from 0.35 ± 0.01 to 0.18 ± 0.02; p < 0.001) (Fig. 7e) in the WT. The activity within the *ArhGAP15*^−/−^ cultures showed a correlation peak that was nearly half that of the WT, indicating loss of synchronicity.

### Working and associative memories are affected in *ArhGAP15* ^−/−^ mice

**Figure 8:**
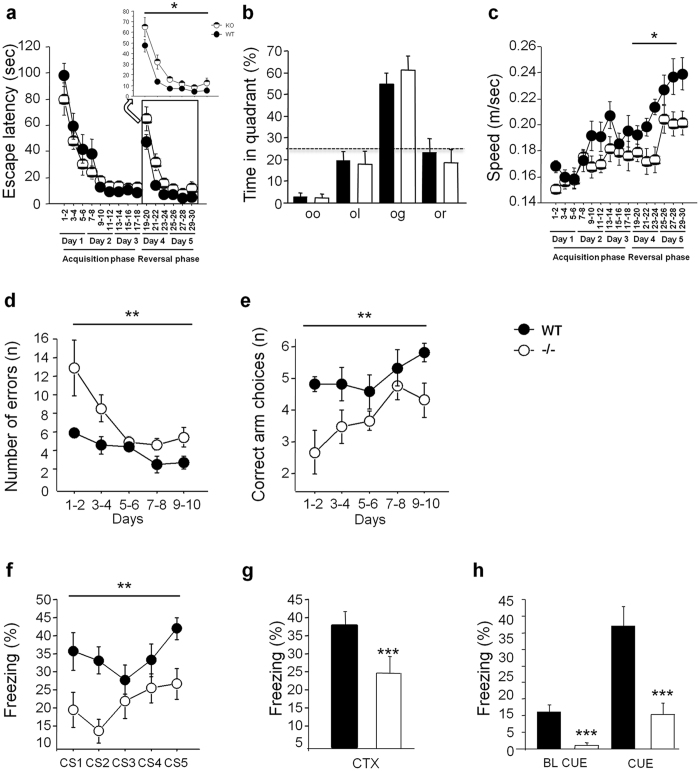
Activity, learning and memory of *ArhGAP15*^−/−^ mice. (**a–c)** *ArhGAP15*^−/−^ mice show deficits in spatial learning, using the hidden platform version of the water maze test. During the acquisition phase the platform was placed always in the same position. On the fourth day the platform was moved to a different position in the reversal phase (2 days of 6 trials per day). In the reversal sessions there were significant differences between WT and *ArhGAP15*^−/−^ in the time required to reach the platform (*) (**a**), in the path travelled (*) and in the speed (*) (**c**). No change is observed in the time spent in each quadrant (**b**). **(d–e)** *ArhGAP15*^−/−^ mice show deficits in short-term working memory and procedural learning. Using the 8-arms radial maze test, *ArhGAP15*^−/−^ mice show a significantly slower acquisition, while the number of visits and the total number of errors (**d**) are higher (**). *ArhGAP15*^−/−^ mice differ significantly in the position of the first repetition (**), e.g. they make less correct arm choices before their first error (**e**). **(f–h)** *ArhGAP15*^−/−^ mice show deficit in associative memory and learning, assessed using an auditory fear-conditioning protocol. The animals exhibit an immobility response (freezing) after exposure to tones (conditioning stimulus, CS) paired with a foot shock (unconditioned stimulus US). During the training phase, freezing reactions was significantly fewer in the *ArhGAP15*^−/−^ (**) (**f**). In the recall session, significant differences were observed between genotypes when 24 hrs later the animals were tested for freezing reactions to the context (***) (**g**) or to the tone (***) (**h**). These results indicate that *ArhGAP15*^−/−^ mice are unpaired with respect to the fear-related memory formation. Experiments were conducted on 10 animals per genotype. *, ** and ***Indicate, respectively, p < 0.05, p < 0.01 and p < 0.001.

We evaluated hippocampus-dependent behaviours in adult *ArhGAP15*^−/−^ mice, compared to WT animals. At first, we determined how *ArhGAP15*^−/−^ mice perform in a spatial memory reference task, using the hidden-platform water maze test. During the acquisition phase, no difference was observed in the time to reach the platform (ANOVA genotype effect: F[1,18] = 1.21, p = 0.286) suggesting that mutant mice have a normal ability to learn and solve the task (Fig. 8a). Accordingly, during the probe trial control and mutant mice showed a higher preference for the trained goal quadrant compared to the averaged time in the three control zones (ANOVA genotype effect: F[1,18] = 0.2, p = 0.7; Fig. 8b). The analysis of the reversal phase revealed a significant difference between genotypes towards longer escape latency (ANOVA genotype effect: F[1,18] = 7.1, p = 0.017; Fig. 8a) and decreased speed (ANOVA genotype effect: F[1,18] = 6.9, p = 0.017; Fig. 8c) in mutant mice. *ArhGAP15*^−/−^ mice showed normal ability to learn this task, as shown by the probe trial in the first day of reversal phase, however during the reversal phase they has difficulties to locate the new platform position.

Next we examined spatial working memory, using the radial maze test. The number of errors declined over the 10 days of training in both groups of mice (ANOVA days: F[4,56] = 4.64, p = 0.003; Fig. 8d). However, in the *ArhGAP15*^−/−^ mice the acquisition was significantly slower than the controls (ANOVA genotype effect: F[1,14] = 12.3, p = 0.0035; genotypes by days interaction: F[1,4] = 1.22, p = 0.31; Fig. 8d). WT and *ArhGAP15*^−/−^ mice differed significantly in the position of the first repetition (ANOVA genotype effect: F[1,14] = 10.8, p = 0.0053; genotypes by days interaction: F[1,4] = 0.71, p = 0.59; Fig. 8e); indeed the *ArhGAP15*^−/−^ mice were barely above chance level performance. This suggests that the *ArhGAP15*^−/−^ mice have defects in procedural learning as well as in working memory.

Finally, we assessed associative learning using the auditory trace fear-conditioning paradigm. *ArhGAP15*^−/−^ mice showed a significantly reduced freezing response compared to controls, in the presence of the CS during conditioning as well as in the subsequent context and tone test sessions, done 24 hrs later (Fig. 8f–h). In *ArhGAP15*^−/−^ mice, the presentation of the CS elicited a freezing response that increased over the five conditioning trials; however this increase in mutant animals was significantly reduced compared to the WT (ANOVA genotype effect: F[1,17] = 11.7, p = 0.003; genotypes by days interaction: F[1,4] = 1.23, p = 0.30; Fig. 8f). Neither genotype groups exhibited freezing in response to the context (test chamber) alone, during the context test sessions (ANOVA genotype effect for CTX: F[1,17] = 5.4, p = 0.03; Fig. 8g), nor when presenting the CS in a new environment during the second half of the tone test (ANOVA genotype effect for CUE: F[1,17] = 16.69, p = 0.0008; Fig. 8h). In conclusion, *ArhGAP15*^−/−^ mice display normal spatial memory but impaired working and associative memory.

## Discussion

We show that in the hippocampus, disruption of the negative Rac1/3 regulator *ArhGAP15* alters neuritogenesis and the balance between excitatory and inhibitory synapses. INs appear most affected, with altered directional migration, reduced number of specific subpopulations (CR+, PV+ and SST+) and reduced synaptogenesis. Consistently, cultured *ArhGAP15*^−/−^ neurons show over-excitation and reduced synchronicity. This endophenotype can be summarized as a global reduction of complexity, with a main impact on the organization and activity of the inhibitory network. This has consequences on hippocampus-dependent cognitive performances.

Although a minority with respect to principal neurons, inhibitory neurons take part in most adult hippocampal functions, from learning/memory and plasticity to emotional value association^41^,^42^. They do so by fine-tuning and synchronizing the flow of information, and by sustaining a delicate excitatory/inhibitory balance within neuronal networks^43^,^44^. The local electrical activity of INs is essential for hippocampal functions; indeed perturbations of IN number and/or activity with consequent enhanced or reduced inhibitory tone contribute to several neurological and cognitive disorders^43^,^44^,^45^,^46^,^47^. Strong support comes from mutant mouse models in which specific subpopulations of hippocampal INs are depleted or functionally inefficient. Such is the case of conditional disruption of *GluR-A* in PV+ hippocampal neurons, leading to imprecise fast-spiking, defective synchronicity and learning/memory deficits^41^. Further support comes from the converse observation: increased GABAergic inhibition in the DG enhances reactivity to novel objects and improves learning and memory^48^. Finally, a role for INs and GABAergic inhibition in ID^43^,^47^,^49^, in models of schizofrenia^45^,^50^, of Down syndrome^49^ and of Rett syndrome^51^,^52^ has been documented.

In the absence of *ArhGAP15* we observe fewer inhibitory synapses on the soma of both CA1–CA3 and DG principal neurons, suggesting a globally reduced inhibition. Concerning excitation, we observe reduced spine density, however associated to unchanged basal electrical properties. Finally the density of excitatory synapses was reduced and increased, respectively, in the CA3 and the CA1–CA2 regions. The scenario (summarized in Fig. 4e) is that of a globally reduced GABAergic inhibition in all regions: in CA1–CA2 and DG this is unbalanced in favour of excitatory input, while in CA3 it appears balanced. We provide evidence that the reduced inhibition prevails over the reduced excitation; in fact primary cultures of *ArhGAP15*^−/−^ hippocampi shows increased firing frequency and burst number. Notably, this condition has been observed in *Rac1N/Rac3* hypomorphic mutant mice^3^,^11^.

To further support this, we find that spontaneous networks of dissociated neurons from *ArhGAP15*^−/−^ hippocampi showed a clear loss of synchronicity. A role for fast-spiking INs to sustain synchronicity and spontaneous EEG oscillations has long been proposed^41^,^53^. Recently, highly connected “hub” neurons have been identified and shown to synchronize the excitatory output of CA1–CA3 region principal neurons^54^. Such “hub” neurons have been further characterized as being fast-spiking GABAergic neurons, of the “basket” morphological type. More specifically, CR+ INs have been shown to play a role in synchronizing other inhibitory INs within the hippocampus^55^, while PV+ neurons control synchronicity in a more general fashion^56^. Since we detect a selective reduction in the number of CR+ and PV+ INs in the absence of *ArhGAP15*, we derive that the altered synchronicity of spontaneously organizing *ArhGAP15*^−/−^ networks can be ascribed to disturbance of these IN types. Notably, a link between the correlation/synchronicity of neuronal hippocampal networks and learning has been previously established^57^, thus we propose that the loss of synchronicity is directly linked to reduced cognitive performances of the *ArhGAP15*^−/−^ animals.

PV+ neurons are receiving increasing attention; they are fast-spiking, exert a potent inhibitory action on the principal neurons and have been implicated in memory formation, retention and recall, as well as in learning^41^,^58^,^59^,^60^,^61^. Recently, the level of PV expression has been correlated with a plastic configuration of the neuronal networks comprising the PV+ neuron^59^,^60^. In these works, a low-PV expression high-plasticity state, versus a high-PV expression low plasticity state, were induced with, respectively, highly enriched environments and fear-conditioning protocols. In *ArhGAP15*^−/−^ mice we observe a reduced number of PV+ neurons in the DG, and defective working and associative memory. It will be crucial to determine if the loss of *ArhGAP15* alters the relative fraction of high- medium- or low-expressing neurons irrespective of previous experiences, and if this is associated to their altered plasticity and/or electrical properties. On the other side, CR+ neurons have also been shown to play a role in long-term potentiation^62^,^63^ and possibly in learning and memory. In the absence of *ArhGAP15* we also noticed a reduced number of CR+ neurons, thus the cognitive deficits of *ArhGAP15*^−/−^ animals are likely to result from the combined impairment of both PV+ and CR+ neuron subtypes. It will important to dissect the specific contribution these IN populations.

During development, INs participate in key aspects of cortical and hippocampal organization ranging from trophic and guidance to pacing the wiring and activity of neuronal networks^64^. While a role of Rac1 for radial migration and axon guidance of cortical excitatory neurons has been well documented^6^,^8^,^36^, our data and evidence from the literature indicate that INs in general are exquisitely vulnerable to changes in the amount/activity of Rac1/Rac3. Indeed, the cellular, electrical and behavioural phenotypes of *ArhGAP15*^−/−^ mice are indicative of a pronounced impairment of GABAergic INs rather than principal neurons, although *ArhGAP15* is expressed by both neuronal types. Furthermore, loss-of-function or dominant-negative mutations of *Rac1* affect mainly hippocampal INs^9^,^10^,^11^,^13^. Finally, in late development, we observed altered migration properties of immature INs, possibly explained by altered control of directionality, as reported for neurotrophils^18^. The endophenotypes affecting INs in the absence of *ArhGAP15,* however, are likely to affect the acquisition of mature functions by principal neurons, directly and indirectly. Our analyses, though, have excluded major deficits of these neurons.

Recent analyses on adult single *Rac1* or *Rac3* mutant mice have indicated specific morphological, functional and cognitive differences, suggesting the existence of specialized gene functions^12^. Furthermore mice with the combined *synapsyn-Cre* conditional loss of *Rac1 (Rac1N*) and loss of *Rac3* show aggravated phenotypes affecting migration, axonogenesis and synaptogenesis of hilar mossy cells^9^,^10^,^13^ and of hippocampal and cortical INs^3^,^11^, as compared to the single mutants. Thus Rac1 and Rac3 are not fully redundant. The activity of Rac1 vs. Rac3 cannot be distinguished at this moment, however we favor the hypothesis that ArhGAP15 acts mainly on Rac1 since this is predominant in the embryonic brain^3^,^6^.

Our results fully support a role for Rac-regulators in hippocampal neuritogenesis and synaptogenesis, whose misregulation leads to cognitive impairment. However, most studies have examined the effect of loss-of-function or dominant-acting mutations; here instead we possibly present the first study showing the effects of a modest increase of Rac1/3 activity caused by loss of a negative controller, hence a condition in which activation is physiological and inactivation is moderately reduced. *ArhGAP15*^−/−^ mice are defective in hippocampus-dependent cognitive functions, such as the spatial learning in water maze test. Specifically, they learn normally but have difficulties to re-localize the platform in the reversal phase, indicating that *ArhGAP15*^−/−^ mice have impaired working memory. Mild memory and learning defects are present in the *Rac1N* mice^9^,^10^,^11^,^12^,^13^ and the described cognitive phenotype is extraordinarily similar to that of *ArhGAP15*^−/−^ mice. Thus we conclude that both hyper- and hypoactivation of Rac1/Rac3 have similar consequences on migration, neuritogenesis and synaptogenesis, and impacts on the maturation of the inhibitory network causing similar cognitive deficits. This suggests that INs are highly sensitive to a precisely and timely regulated Rac1/3, exerted by the numerous regulators expressed in developing neurons.

At the cellular level, while Rac1 is required for neuritogenesis and synaptogenesis, our data indicate that its hyperactivation has consequences similar to its loss. There are instances in which loss or constitutively-active (CA) GTPases result in similar defects of neuronal development. For example, radial migration of cortical progenitors is equally affected by electroporating dominant-negative (or shRNA) or CA Rac1^14^,^65^,^66^,^67^. Moreover, while active Rac1 and cdc42 promote dendrite arborization and spine formation, at least *in vitro*, nonetheless CA cdc42 decreased dendritic arborization^68^. In our view and in the view of others (reviewed in ref. 6), increased or decreased Rac1 activity may alter actin dynamic in complex ways as to prevent normal elongation/branching of the actin filaments and network at the growth cone. Thus, the balanced activity, e.g. a correct amount and timing of activation versus inactivation of each GTPase, is the key physiological regulation.

## Materials and Methods

### Mouse strains and animal procedures

All animals were maintained according to institutional animal welfare guidelines and legislation, approved by the local Animal Ethics Committee and the Ministry of Health. The *ArhGAP15*^−/−^ mouse strain has been previously described^18^. The targeting vector was prepared by inserting a *lacZ* reporter cDNA and disrupting the first exon. Thus *lacZ* expression should recapitulate endogenous gene expression. The animals were maintained in a mixed C57/BL6:DBA genetic background. The mice were overall normal, could feed and mate at regular rates, and did not show evident neurological of motor impairments.

For the collection of embryos, adult (P60) males and females were mated overnight, and the next morning the females were examined for the presence of a vaginal plug. The day of the plug was considered day 0.5 of embryonic development (E0.5). Embryos were obtained by Caesarean section from anesthetized pregnant dams. Extra-embryonic tissues were used for genotyping by PCR.

### Brain preparation for histological analyses

WT, *ArhGAP15*^+*/−*^ or ^−/−^ females were mated with WT, heterozygous or homozygous males to obtain the mutant and the control animals. Embryos were collected by caesarean section at E15.5–E18.5. Embryonic heads were fixed in 4% paraformaldehyde (PFA) pH 7.0 overnight, washed in 5 mM sodium phosphate-buffered 0.9% saline, pH 7.4 (PBS), equilibrated in 30% sucrose in PBS for cryoprotection, embedded in OCT, frozen and stored at −80 °C. For immunohistochemistry and *in situ* hybridization, cryostatic sections were prepared (11 μm-thick) and collected on super-adhesive glass slides. For immunofluorescence, sections (25 μm-thick) were collected and stored in PBS at −20 °C. Adult WT and *ArhGAP15*^−/−^ mice were deeply anesthetized with Avertin (30 μl pure Avertin in 400 μl PBS each animal), transcardially perfused with 30 ml of PBS and then with 50 ml of 4% PFA. Brains were dissected, post-fixed in 4% PFA, washed in PBS and kept in a solution of 30% sucrose in PBS for cryoprotection. The OCT blocks were sectioned (25 μm-thick) with a cryostat; sections were collected in PBS in multi-well plates and stored at −20 until used for immunostaining.

### Immunostaining

Sections were incubated at 4 °C overnight in a solution containing the primary antibody (a detailed list is provided as Supplementary Methods) in 0.5% Tween-20 and 2.5% Bovine Serum Albumin (BSA) in PBS, followed by three rinses of PBS for 10 min. The sections were then incubated for 1 hr with the secondary antibody at RT in a solution of PBS containing 0.5% Tween 20 and 2.5% BSA, rinsed with PBS twice, then incubated 2 hrs at RT with ABC solution (Vector Laboratories, Burlingame, CA). Finally sections were reacted with Dako Cytomation Liquid DAB+ Substrate Chromogen System (Dako, North America Inc.). Sections were mounted onto coated super-adhesive glass slides and covered with Mowiol for microscopic observation. For immunofluorescence, sections were incubated with fluorochrome-labelled secondary antibodies under the same conditions, then incubated with DAPI (1/500) for 15 min at RT before mounting.

For free-floating immunostaining, the sections were blocked in 10% Normal Goat Serum and 4% BSA in 0.5% Triton X-100 in PBS for 1 hr at room temperature (RT), and subsequently incubated with primary antibodies in blocking solution (as above), overnight at 4 °C. The sections were then were washed in 0.1% Triton X-100 in PBS (3 washes, 10 minutes each), incubated with the secondary antibody diluted in PBS, for 2 hrs at RT, washed three times in PBS and incubated with DAPI (1/500) for 10 minutes at RT. Sections were then mounted on glass slides, covered with Mowiol and examined.

For photodocumentation, we used either a Zeiss Observer-Z1 fluorescent microscope equipped with Apotome system, or a Leica SP5 confocal microscope with Z-sections of 0.5 μm. Raw images were digitally processed to normalize the background and optimize the contrast, with Photoshop (Adobe).

### *In situ* hybridization followed by immunostaining

*ArhGAP15* adult mice were anesthetized, cardially perfused with 4% PFA, the brains were post-fixed overnight in PFA, rinsed in RNAse-free PBS, cryoprotected in 30% sucrose for 24 hrs, embedded in OCT, frozen and sectioned at 20 μm thickness. Hybridization was performed with DIG-labeled riboprobes corresponding to the antisense sequence of the murine *ArhGAP15* cDNA and the *lacZ*. Sections were permeabilized with 3 μg/ml proteinase K, washed in PBS, and acetylated with 1.3% triethanolamine and 0.25% acetic anhydrate at RT. Sections were prehybridized in 50% formamide at 60 °C, hybridized with the DIG-labeled probes for 16 hrs, washed, incubated with an anti-DIG-AP antibody (Roche), and developed with NBT-BCIP (Sigma). Sections were mounted transiently, photographed, then immunostained with anti-GAD67 antibody (as above), mounted and re-photographed.

### Western blot and Rac activity assays

Cells or tissues were lysed in 100 μl of loading buffer (2% sodium dodecyl sulfate, 30% glycerol, 300 mM β-mercaptoethanol, 100 mM Tris-HCl pH 6.8): extracts were separated on SDS-10% polyacrylamide gels, transferred and incubated with the relative antibodies and developed according to the manufacturer’s instructions (GeneSpin). An anti-ArhGAP15 monoclonal antibody was also used, this was raised in mouse against a GST-fusion protein of an ArhGAP15 peptide spanning from aminoacids 220–320. For loading control, an anti-actin mouse monoclonal antibody was used (from Sigma).

Rac1/Rac3 activity was measured by pull-down assay, as described^18^. Detailed protocol in Supplementary Methods. The clone 23A8 mouse anti-Rac1 antibody was used (Upstate Biotech, used 1:2000) which, however, also recognizes Rac3 in Western blot analyses (data not shown). The amount of GTP-bound Rac1/Rac3 was also determined with the G-LISA assay (Cytoskeleton, Inc Denver, CO, USA), as previously described^69^. This assay has not been tested for specificity against Rac3.

### Primary cultures of embryonic neurons and analysis of neuritogenesis and spine density

Primary cultures were established either from the hippocampus primordium, at the age E17.5, or from the MGE, at the age E14.5. In both cases, heads were dissected in sterile conditions in Leibovitz’s L-15 Medium (Gibco, Life Technologies), hippocampi or MGEs were dissected, deprived of the meninges and dissociated in Neurobasal medium, supplemented with Glutamine 1/100, B27 1/50 and Gentamicine. At first, mechanical shearing was used, then trypsin was added for 15 min. at 37 °C, followed by centrifugation and resuspension in medium. Cell number was determined by Countess (Life Technologies) and 7 × 10^6^ cells from each pool plated on glass coverslips precoated with poly-L-lysine (1 mg/ml; Sigma; 1/10 dilution) and allowed to adhere. The medium was replaced with fresh medium, neurons were incubated for a total of 7 days at 37 °C in 5% CO_2_ atmosphere. At 5 DIV, neurons were transfected with 3 μg of PGK-eGFP expression vector, using Lipofectamine LTX with Plus Reagent (Life Technology). At the end of the culture time, cells were washed with PBS, fixed for 20 min. with 4% PFA and washed in PBS. Coverslips with adhering neurons were laid on slides, mounted with Mowiol. Fluorescence images were acquired using an inverted microscope (Axio Observed Z1, Zeiss) with ApoTome system. Images were digitally captured using a cooled 16-bit camera (Axio MRM, Zeiss) with Axio Vision Release 4.7.1 software. For the analyses of neuritic length, arborisation and complexity (Sholl analysis) see Supplementary Methods.

For the analyses of spine density and size, dissociated rat hippocampal neurons were plated at 75,000/well and maintained as previously described^70^. At 8 DIV, cultured neurons were infected with anti-*ArhGAP15* shRNA or with the control (scrambled sequence) shRNA vector, previously used and validated^18^, fixed and examined by fluorescent microscopy at 18 DIV as described^70^.

All statistical analyses were done with the Student’s T-test.

### Assay of migration efficiency from hippocampal primordia

Explant cultures were established from hippocampi of E17.5 embryos. After quick dissection, tissues were placed in ice-cold undiluted growth factor-depleted Matrigel (Becton-Dickinson, North Ryde, Australia, www.bd.com), laid in plastic dishes and overlaid with Neurobasal medium supplemented with B27 (Invitrogen) and 0.5 mM glutamine, and maintained 3 DIV. The results were quantified by measuring, for both genotypes: (a) the area occupied by migrating neurons (sq μm) over the corresponding explant perimeter (μm), expressed in μm; (b) the median and maximal distance travelled by migrating cells, expressed in μm. A minimum of 150 cells were examined in each case, statistical analyses were done with T-test. One test culture from both genotypes was fixed and immunostained with ant-GAD67.

### Electrical recording from primary cultures

WT and *ArhGAP15*^−/−^ hippocampi from E17.5 embryos were enzymatically dissociated and plated at density 1200 cells/mm^2^ on poly-L-lysin/laminin coated MEA devices, and maintained for up to 18 DIV in neurobasal medium supplemented with 1% Penn/Strep, 1% Glutamine, 2.5% FBD, 2% B-27 neurobasal, in standard conditions. Recordings were carried out for 90 secs by MEA-MultiChannel System (MCS, Reutlingen Germany), with parameters and procedures detailed in Supplementary Methods. Statistical analyses were done as described in a previous report^40^.

### Learning and memory assessment

Ten WT and ten *ArhGAp15*^−/−^ males, at the age P60, were used for behaviour, learning and memory tests. The animals were housed in groups of five each in a standard home cage with food and water *ad libitum* and an inverted light/dark cycle (lights on at 8:00 pm). Statistical analyses were done with ANOVA. Detailed procedures are provided as Supplementary Methods.

## Additional Information

**How to cite this article**: Zamboni, V. *et al*. Disruption of *ArhGAP15* results in hyperactive Rac1, affects the architecture and function of hippocampal inhibitory neurons and causes cognitive deficits. *Sci. Rep.* **6**, 34877; doi: 10.1038/srep34877 (2016).

## Supplementary Material

Supplementary Information
