# Supplementary material for: Disruption of ArhGAP15 results in hyperactive Rac1, affects the architecture and function of hippocampal inhibitory neurons and causes cognitive deficits
Source: Sci Rep. 2016 Oct 7;6:34877. doi: 10.1038/srep34877 (PMC5054378; doi:10.1038/srep34877)
Supplement: Supplementary Information [file srep34877-s1.pdf]

Disruption of *ArhGAP15* results in hyperactive Rac1, affects the architecture and function of hippocampal inhibitory neurons and causes cognitive deficits

Valentina Zamboni, Maria Armentano, Gabriella Sarò, Elisa Ciraolo, Alessandra Ghigo, Giulia Germena, Alessandro Umbach, Pamela Valnegri, Maria Passafaro, Valentina Carabelli, Daniela Gavello, Veronica Bianchi, Patrizia D'Adamo, Ivan de Curtis, Nadia El-Assawi, Alessandro Mauro, Lorenzo Priano, Nicola Ferri, Emilio Hirsch and Giorgio R. Merlo

Supplementary Methods

X-gal staining

For X-gal staining, *ArhGAP15*$^{+/-}$ and $^{-/-}$ embryonic and adult brains were thick-sectioned (150-200 μm) by vibratome, fixed shortly (20 min) with 4% PFA, rinsed in PBS and stained with a solution containing 0.2 M $K_3Fe(CN)_6$, 1 M $MgCl_2$, 0.2 M $K_4Fe(CN)_6$, 25% NP-40, and 1 mg/ml of Xgal. The reaction was continued for 3-10 hrs, depending on signal intensity, at 32°C in dark. Subsequent to Xgal staining, sections were mounted on slides.

Antibodies used for immunostaining

The following primary antibodies were used: mouse anti-NeuN (MAb377, clone A60, Millipore, used 1:100), rabbit anti-Glutamic Acid Decarboxylase (GAD67, Millipore, used 1/1000), rabbit anti-Calretinin (CR, Swant, used 1/2500), rabbit monoclonal anti-Parvalbumin (PV, Swant, used 1/5000), rabbit anti-Calbindin (CB, Swant, used 1:2000), rabbit anti-Somatostatin (SST, Millipore, used 1/1000), rabbit anti-Vescicular GABA Transporter (VGAT, Synaptic System, used 1/1000) and rabbit anti-Glial Fibrillar Acidi Protein (GFAP, DAKO, used 1/500). We also used mouse anti-βGalactosidase (βGal, Zymed, used 1/1000). Secondary antibodies were biotinylated anti-rabbit and anti-mouse IgG (Jackson Laboratories, West Grove, PA, used 1/1000), for immunoperoxydase, or Alexa Fluor 488 donkey anti-rabbit IgG and AlexaFluor 568 goat anti-mouse IgG (Invitrogen, used at 1/400) for immunofluorescence.

Pull-down assay for Rac1/Rac3 activity

Rac1/Rac3 activity was measured by pull-down assay. A glutathione-S-transferase-PAK-CD (PAK-CRIB domain) fusion protein, containing the Rac binding region from human PAK1, was used to determine Rac activity. Lysates of the embryonic cortices were centrifuged at 4°C for 10 min at 13,000 RPM, and the supernatant was incubated with glutathione S-transferase PAK glutathione-

coupled Sepharose 4B beads (GE Healthcare) for 30 min. at 4°C. Proteins bound to the beads were washed 3 times in lysis buffer, and then quantified by polyacrylamide gel electrophoresis and Western Blot analysis using a mouse anti-Rac1 antibody (clone 23A8, Upstate Biotech., used 1:2000) which, however, also recognizes Rac3 in Western blot analyses (data not shown). Images were quantified by densitometric analysis using Quantity One software (BioRad, CA, USA).

Analysis of neuritogenesis and neuronal morphology

Neuritogenesis was assayed by measuring the following parameters: the length of the longest neurite, the number and distribution of branches (indicating the arborization rate) and the number of secondary neuritis departing from the main neurite of at least 150 GFP+ neurons per genotype. To perform this analysis, we used basic functions of ImageJ software. In addition, the global arborization and complexity of each neuron was assessed by “Sholl Analysis”, done on a 100 GFP+ neurons per genotype: this computer-assisted method uses a series of concentric circles around the soma of each neuron, and determines how many times its neurites intersect each of these circles (plugin ImageJ), expressed as a mean value.

For neuronal morphology, a minimum of 150 GFP+ neurons were photographed and classified as unipolar, bipolar and multipolar, according to the number of visible primary neuritis departing from the soma. The analysis was done using the CellCounter plugin of ImageJ. Finaly, for the multipolar neurons the number of primary neurites was also counted.

All statistical analyses were done with the Student’s T-test.

Electrical recording from primary cultures

WT and *ArhGAP15*$^{-/-}$ hippocampi (E18) were enzymatically dissociated and plated at density 1200 cells/mm^2 on poly-L-lysin/laminin coated MEA devices, and maintained for up to 18 DIV in neurobasal medium supplemented with 1% Penn/Strep, 1% Glutamine, 2.5% FBD, 2% B-27

neurobasal, in a humidified 5% CO_2 atmosphere at 37°C. One third of the culture medium was changed once a week.

Multisite extracellular recordings were carried out starting at 7 DIV and up to 18 DIV, by means of the MEA-MultiChannel System (MCS, Reutlingen Germany). Data acquisition was controlled through the MC_Rack software (MultiChannel System, Reutlingen Germany) setting the threshold for spike detection at -15 μV and sampling at 10 kHz. Each recording lasted for 90 seconds. Burst analysis was performed using the Neuroexplorer software (Nex Technologies, Littleton MA USA) after spike sorting. A burst is defined as a group of spikes with decreasing amplitude (Bean BP, 2007), thus we set a threshold of at least 3 spikes and a minimum of 10 ms duration. We set interval algorithm specifications such as maximum interval to start burst (0.17 sec) and maximum interval to end burst (0.3 sec) recorded in 0.02 s bins. Burst analysis was carried out by measuring mean frequency and number of bursts.

To examine synchronicity, cross-correlation probability vs. time diagrams were constructed by means of the Neuroexplorer software, using ± 0.5 s and ± 3.5 s and 5 ms bin size. Data are expressed as means ± S.E.M. and statistical significance was calculated with the Student's unpaired T-test.

Learning and memory tests

The dark-light box test for anxiety-like behaviour was carried out using a 20 x 30 x 20 cm chamber with transparent Perspex walls and open top was connected to a 20 x 15 x 20 cm plastic dark box that was completely enclosed except for a 7.5 x 7.5-cm connection door. The transparent chamber was illuminated by direct light (500 lux). Each mouse was released in the middle of the lit transparent chamber and tracked for 5 min.

For the emergence test, frames of non-reflective black Perspex walls (37-cm high) were used to partition a round open field arena (diameter of 150 cm and 35-cm high walls) into four square 50×50 cm arenas, allowing for concurrent tracking of four animals at one time. Illumination in the

room was provided by indirect diffuse light (4x40-W bulbs, 12 lx). The day before the test a plastic box (12x8x4 cm with an aperture of 8x4 cm) was inserted in each mouse’s home cage. The same box was then placed in a corner of the arena the testing day. Each box was placed 5 cm from the central walls’ corner, with the opening facing the centre of the arena. The mice were released in the centre and tracked for 30 min.

For the water maze test, mice were trained in a circular pool (150 cm diameter and 50 cm height) according to standardized protocols (Wolfer DP and HP Lipp, 1992). The wire-mesh platform was 14x14 cm. In the hidden-platform version of the water maze, mice had to locate a hidden platform in a fixed position. The test included an acquisition phase (18 trials, 6/day, inter-trial time 30–40 min) followed by a reversal phase during which the platform was moved to the opposite position (12 trials, 6/day). For the analysis the trials were averaged in blocks of two trials. The following measures were calculated to assess acquisition: escape latency, swim speed, time floating, wall hugging and the percentage of time in the current quadrant goal (excluding episodes of floating). Spatial selectivity during the probe trial was quantified using the following parameters: percentage of time in the trained quadrant, percentage of time in a circular target zone comprising 1/8 of the pool surface and the annulus crossings. The following measures were calculated to assess platform reversal learning: escape latency and the percentage of time in current quadrant goal (excluding episodes of floating).

For the eight-arms radial maze test, the apparatus consisted of eight arms (38 cm long, 7 cm wide) extending from an octagonal centre platform (diameter 18.5 cm) with 5-cm transparent plastic walls. The distance from the platform centre to the end of each arm was 47 cm. At the end of each arm is present a cup with a food pellet. Food-deprived mice (maintained at 85% of their free-feeding weight) were were placed in the centre platform and allowed to collect pellets placed at the end of each arm for 10 min. The animals were adapted to the maze for 1 day and then tested for 10 days. For each trial, the total number of arm choices, number of correct choices before the first error, total numbers of errors were recorded.

The trace fear conditioning tests were performed by placing the mice in an opaque conditioning chamber (LxWxH: 25x17x23 cm) with a grid floor through which scrambled foot shocks could be delivered as unconditioned stimuli (US; 0.26 mA average intensity). The chamber was placed into a dimly lit (<5 lux) sound attenuating box (background noise level 55 dB), and a speaker on top of the chamber allowed to deliver sounds as conditioning stimuli (CS; 2000 Hz). All mice were pre-exposed to the test chamber for 10 min on the day preceding conditioning. On day two, the training session, 5 trials were presented: the first trial was preceded by a 60-sec baseline period (no tone), followed by the presentation of a 15-sec tone (CS, 85 dB 2000 Hz) and 15-seconds later the CS offset (which was the trace) by a 2-seconds foot-shock (US, 0.2 mA). After US offset there was a 60-sec interval here regarded as inter trial interval (ITI) before the next trial begun. Twenty-four hrs after fear conditioning, mice were placed again in the conditioning box and scored for freezing behaviour in both the context test (2 min without CS "context induced freezing") and subsequently in the cue test (new environment, 2 min divided in: first min as baseline and second min with CS "Cue induced freezing"). During the test, animals were continuously video-tracked using the ANY-maze system (Anymaze, Stoelting Co, Wood Dale, IL, USA, www.anymaze.com). The frequency of freezing (absence of movements except respiration) was continuously recorded.

During the tests, animals were video-tracked using the EthoVision 2.3 system (Noldus Information Technology, Wageningen, the Netherlands, http://www.noldus.com) using an image frequency of 4.2/s. Raw data were transferred to Wintrack 2.4 (http://www.dpwolfer.ch/wintrack) (Wolfer and Lipp 1992)for off-line analysis. Statistical computations were done using Statview 5.0 (SAS Institute, Cary, NC, USA, www.statview.com). ANOVA was used to compare genotype effect. Repeated ANOVA was used to compare genotype effect across different sessions and to check session dependence of mutation effect, "session" was included as an additional between-subject ANOVA factor.

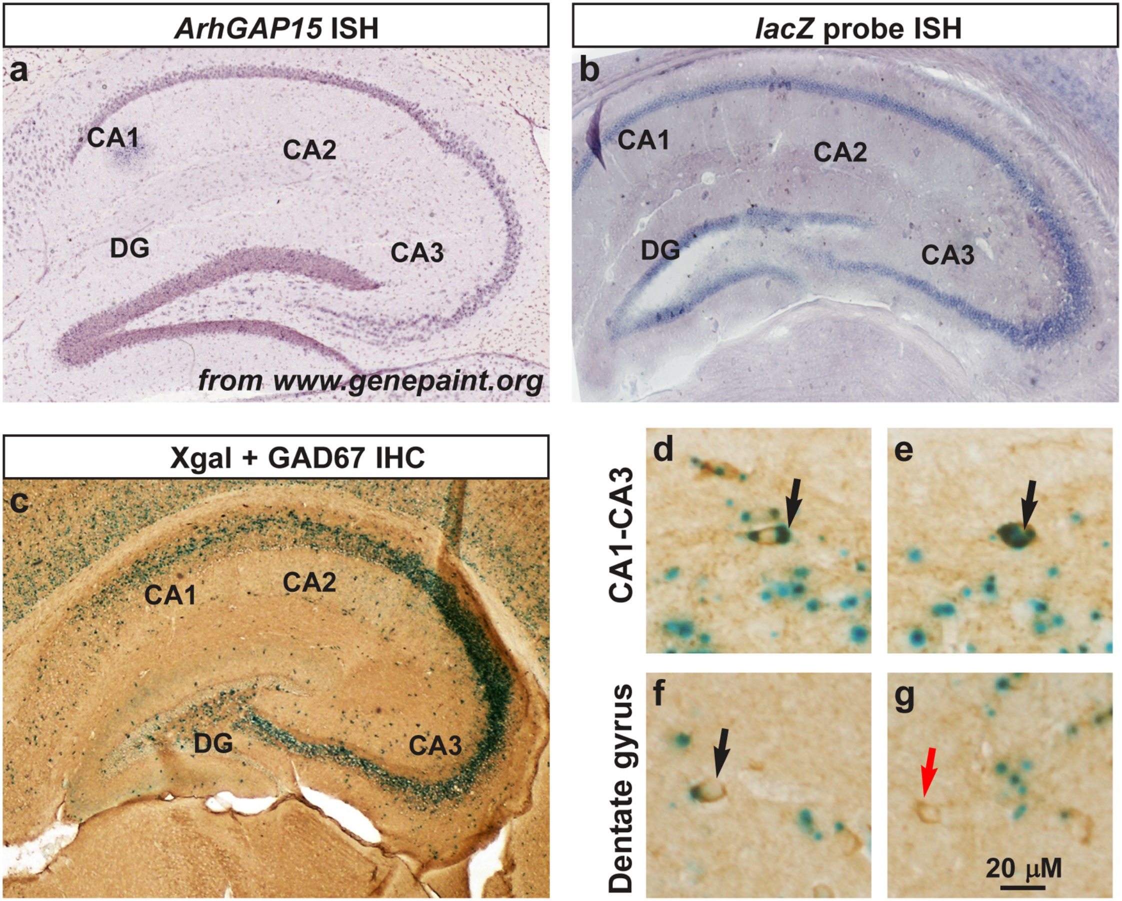

Supplementary Figure S1

a. Scheme of the general organization of the hippocampus. **b**. *in situ* hybridization of a coronal section of adult hippocampus from *ArhGAP15*$^{+/-}$ mice, to detect the *lacZ* mRNA. **c-g**. Coronal sections of *ArhGAP15*$^{+/-}$ adult hippocampus, stained with Xgal and immuno-stained with anti-GAD67, on the same section. On the right (d-g), higher magnification of double positive (Xgal + GAD67, black arrows) or single positive (only GAD67, red arrow) neurons within the DG. Scale bar is reported in panel g.

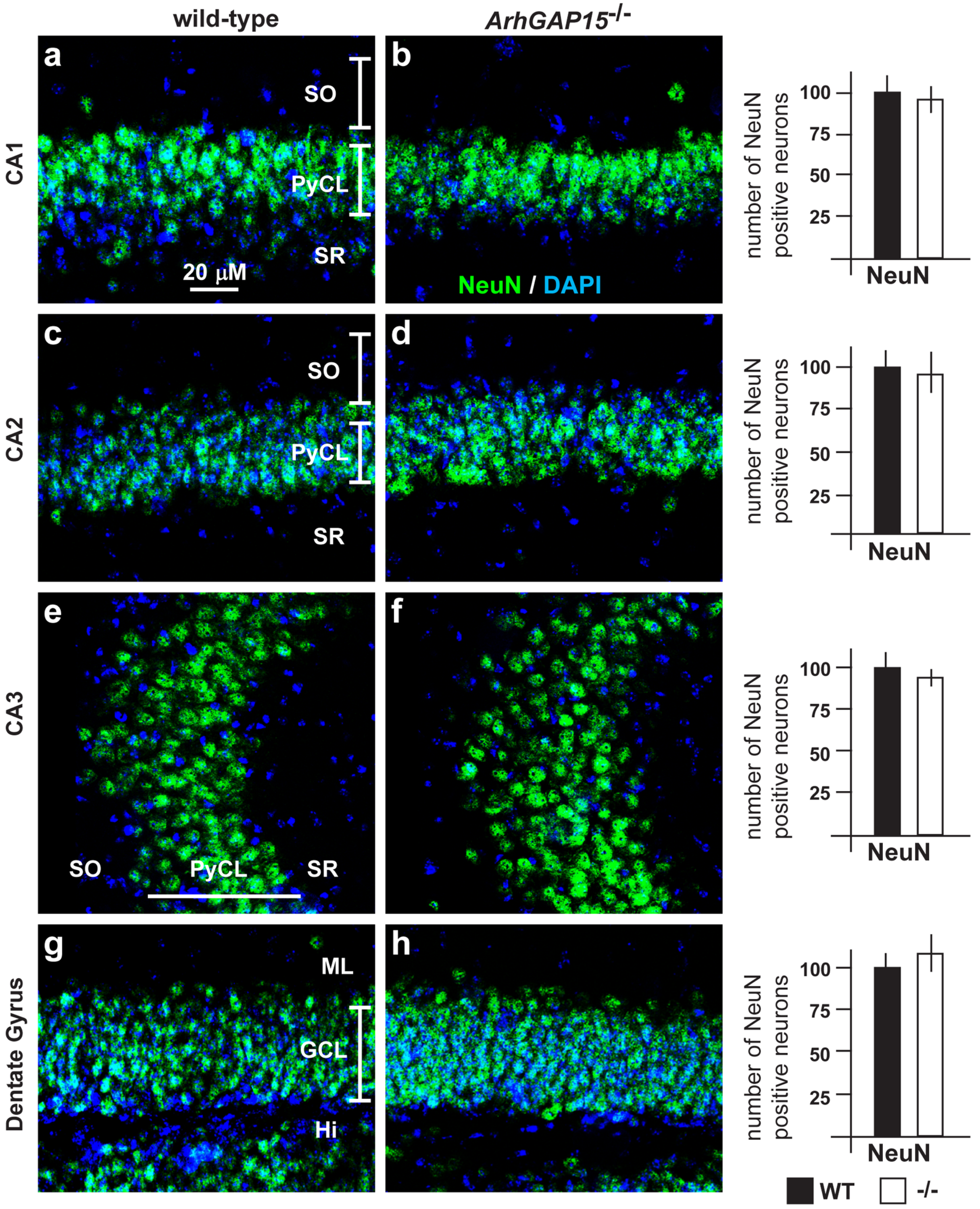

Supplementary Figure S2

Principal neurons in the hippocampus. **a-h.** Representative immunofuorescent staining of WT (left) and *ArhGAP15-/-* (right) adult hippocampi CA1 region (a,b), the CA2 region (c,d), the CA3 region (e,f), and the DG (g,h). NeuN+ neurons are stained in green, nuclei are stained with DAPI (blue). Histograms on the left report the number of NeuN+ cells in the two genotypes. Results are from three animals for each genotype. WT is set = 100%. Cell layers are indicated. GCL, Granule Cell Layer; Hi, Hilus; ML, Molecular Layer; SO, Stratum Oriens; SR, Stratum Radiatum; PyCL, Pyramidal Cell Layer. Scale bar is shown in panel a.

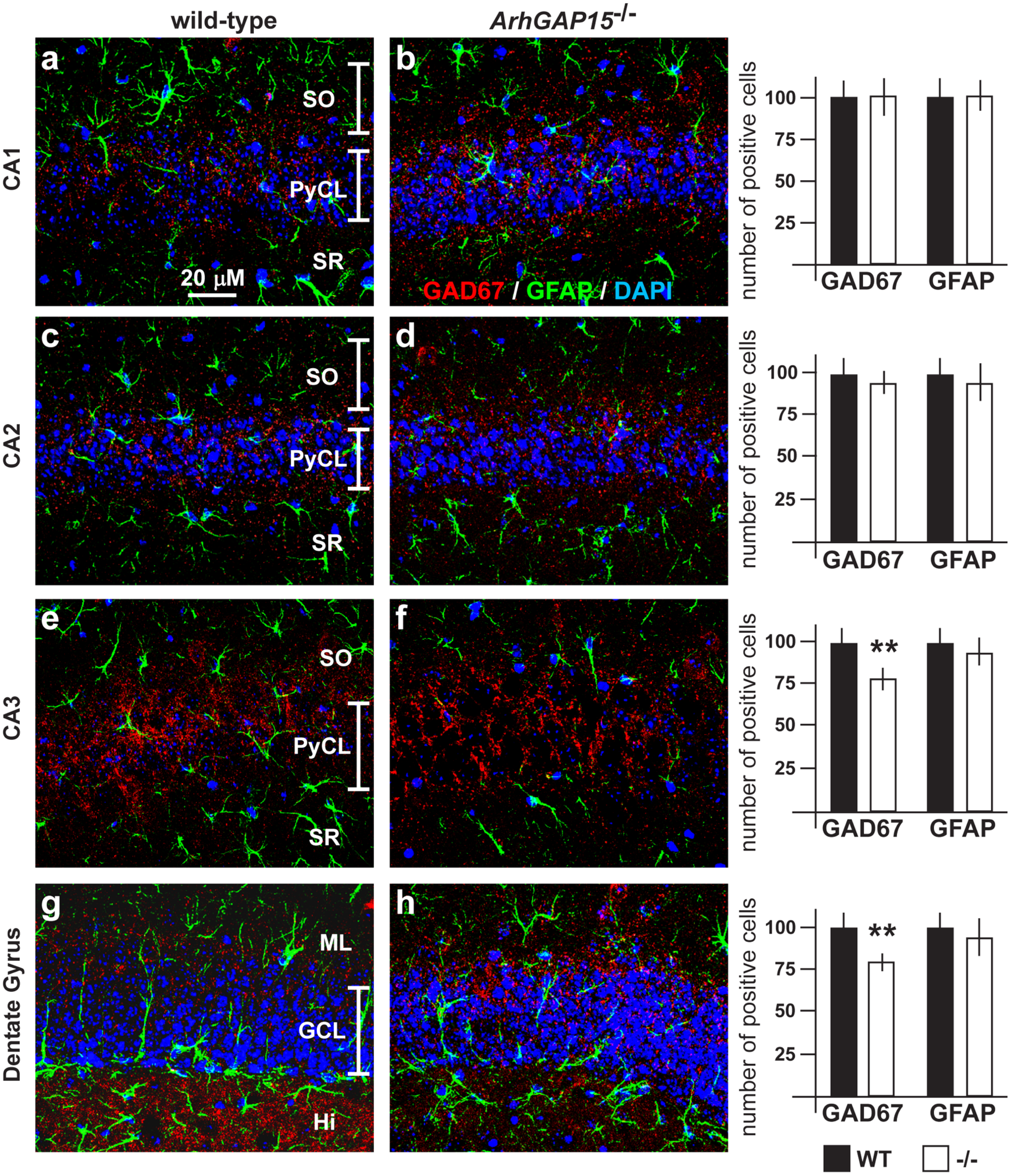

Supplementary Figure S3

Inhibitory neurons (INs) and astrocytes in the hippocampus. **a-h.** Representative immunofuorescent staining of WT (left) and *ArhGAP15-/-* (right) adult hippocampi CA1 region (a,b), CA2 region (c,d), CA3 region (e,f), and the DG (g,h). GAD67 is stained in red, GFAP is stained in green. Nuclei are counterstained with DAPI (blue). Histograms on the left report the number of GAD67+ and GFAP+ cells in the two genotypes. Results are from three animals for each genotype. WT is set = 100%. Cell layers are indicated. GCL, Granule Cell Layer; Hi, Hilus; ML, Molecular Layer; SO, Stratum Oriens; SR, Stratum Radiatum; PyCL, Pyramidal Cell Layer. ** indicate statistical significance ($p<0.01$). Scale bar is reported in panel a.

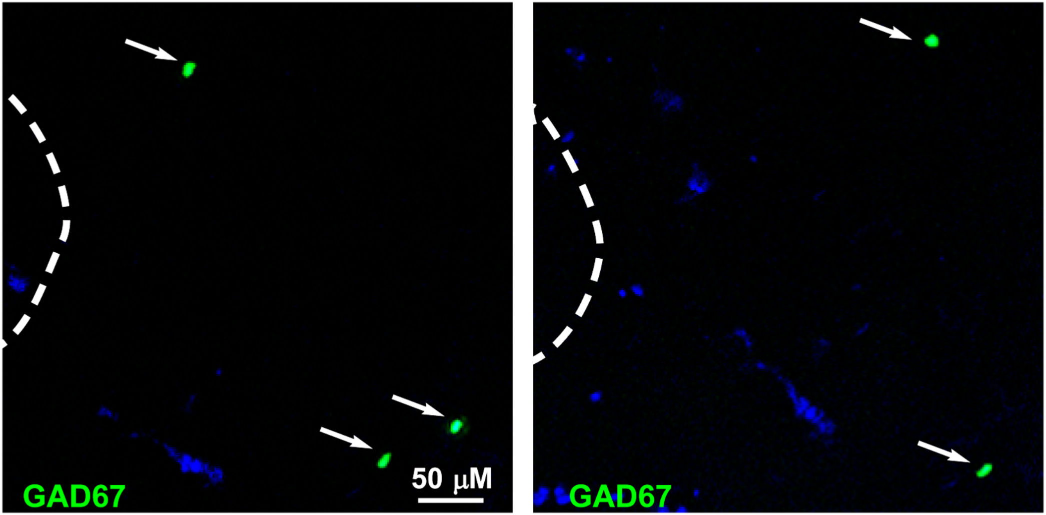

Supplementary Figure S4

Immunostaining of cultured explants of embryonic hippocampi (E17.5) for GAD67, after 3 DIV, to confirm the presence of early differentiating inhibitory neurons among the out-migrating cells. Two representative images showing GAD67+ cells (green fluorescence, indicated with white arrows). The position of the explant is indicated with dashed white lines. A total of eight cultures were examined. It is estimated that about 15% of all out-migrating neurons are GAD67+, and that nearly all the longest migrating ones are GAD67+. Scale bar is reported in the left panel.

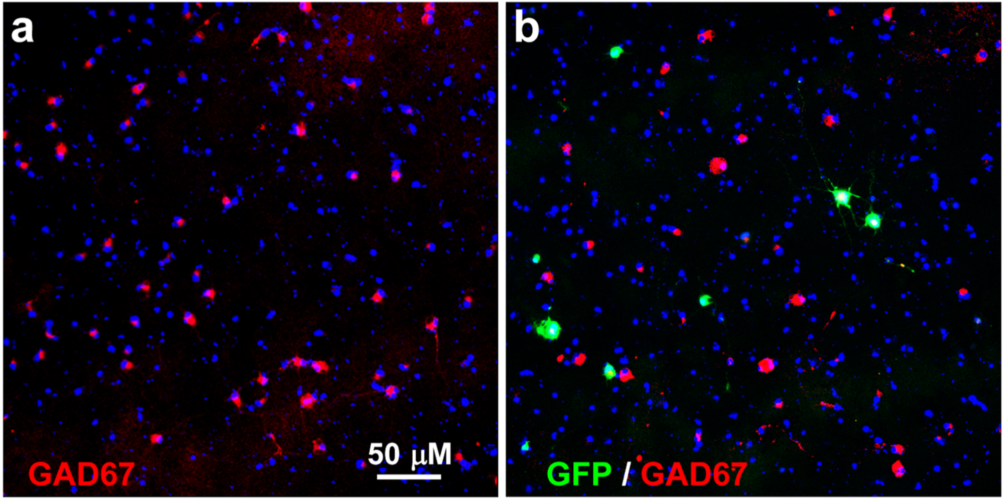

Supplementary Figure S5

Immunostaining of 7 DIV primary cultures of dissociated cells from the MGE of WT embryos, at the age E14.5, for GAD67 (a) and for GAD67 + GFP fluorescence derived from the transfected expression vector (b). A total of seven cultures were examined. Scale bar is reported in panel a. The results confirm that the majority of cells present in the culture are inhibitory neurons, and confirm that the GFP transfected cells are all interneurons.

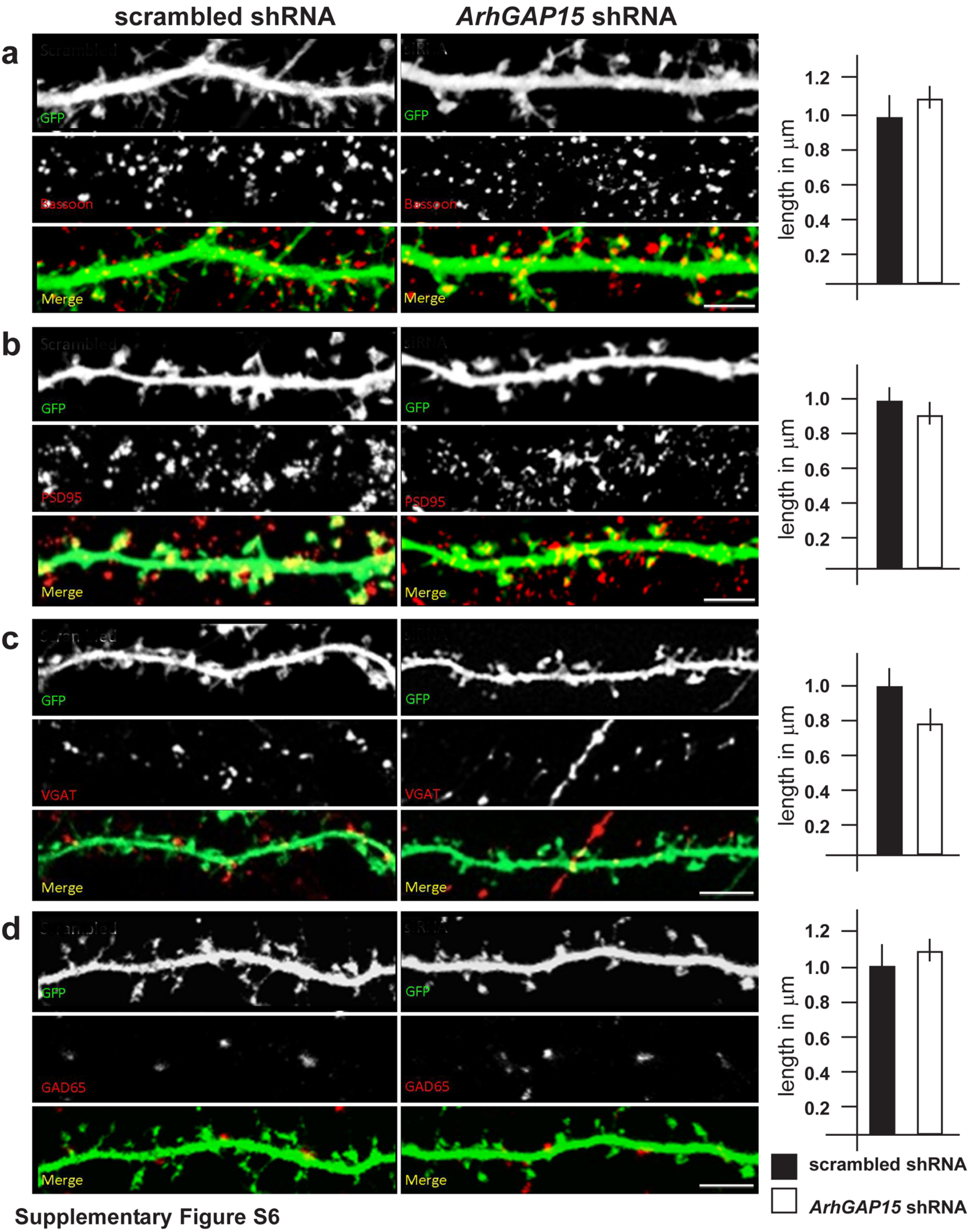

Supplementary Figure S6

Control staining of hippocampal neuron dendrites, in which the endogenous *ArhGAP15* has been silenced. Cells are stained with antibodies recognizing the following synaptic proteins: **a**, Bassoon; **b,** PSD95; **c**, VGAT (inhibitory pre-synaptic) and **d**, GAD65 (inhibitory pre-synaptic). The transfected neurons (control or *ArhGAP15* depleted) are labelled in green. For each marker, the merge with the GFP reporter is shown below. On the right, quantification of the number of positive synapses, in control (solid bars) and silenced (open bars) cultures. A total of 40 neurons in three experiments were examined. No significant differences were observed with any of these markers, indicating that the general organization and neurotransmitter specificity of the synapses was not changed by depleting *ArhGAP15*. Scale bars (0.1 µM) are shown in each panel, lower right corner.

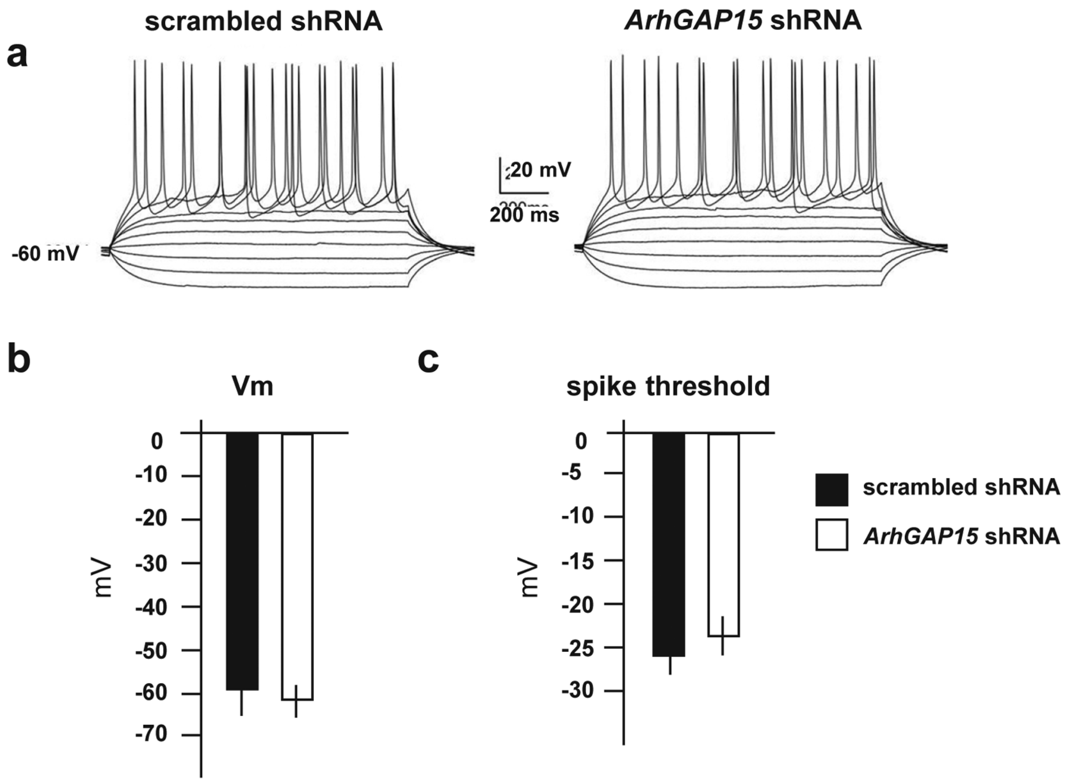

Supplementary Figure S7
Membrane potential and excitation threshold of rat hippocampal neurons, in which ArhGAP15 was silenced. Single-cell recording of GFP+ control (N=20) and silenced (N=21) neurons. a. Membrane potential and threshold for eliciting a response. The time and amplitude scale is indicated. b. Quantification of the results shown in panel a. No significant difference was observed.
